# Recent advances in interstitial fluid dynamics for diagnostic, targeted drug delivery and precision medicine applications

**DOI:** 10.1007/s13346-026-02106-9

**Published:** 2026-03-26

**Authors:** Khonzisizwe Somandi, Hlumelo Kulati, Darin E. Holman, Ameerah-Imaan Abrahams, Mpho P. Ngoepe, Marshall Keyster, Yahya E. Choonara

**Affiliations:** 1 Wits Advanced Drug Delivery Platform Research Unit, Department of Pharmacy and Pharmacology, School of Therapeutic Science, Faculty of Health Sciences, University of the Witwatersrand, 7 York Road, Parktown, Johannesburg, 2193 South Africa; 2 Department of Physiology, Faculty of Science, Nelson Mandela University, University Way, Summerstrand, 6019 Gqeberha South Africa; 3 Environmental Biotechnology Laboratory, Department of Biotechnology, University of the Western Cape, Robert Sobukwe Road, Bellville, 7530 South Africa; 4 DSI-Mandela Nanomedicine Platform, Nelson Mandela University, Gqeberha, 6059 South Africa

**Keywords:** Interstitial Fluid, Diagnostics, Precision Medicine, Drug Delivery

## Abstract

Interstitial fluid (ISF), the extracellular fluid surrounding tissue cells, has emerged as a promising biofluid for biomarker detection and localized physiological monitoring. Although its clinical use remains limited compared with blood or urine, advances in minimally invasive extraction technologies have expanded research interest in its diagnostic and therapeutic potential. ISF shares biochemical similarities with blood but is more accessible through minimally invasive techniques, offers localized biomarker concentrations, and avoids clotting issues. Recent research has demonstrated that ISF contains a rich profile of biomolecules, including proteins, nucleic acids, metabolites, and cytokines, that are valuable for diagnosing and managing a variety of conditions such as infections, metabolic disorders, cancers, and neurological diseases. Advances in microneedle (MN) technology, wearable biosensors, and minimally invasive extraction methods have enabled the efficient sampling and analysis of ISF, bringing its clinical applications closer to reality. Beyond diagnostics, ISF is now being leveraged in therapeutic applications, particularly in precision medicine and drug delivery. Novel delivery strategies such as MNs, convection-enhanced delivery (CED), electroosmotic flow, and fiber-optic theranostics use ISF as both a conduit and a target environment, especially for conditions like solid tumors, where interstitial fluid pressure (IFP) plays a critical role in treatment efficacy. Simulated ISF models are also being developed to facilitate preclinical drug testing and improve translational success. This review emphasizes the mechanistic physiology of ISF from both human and preclinical/animal models, including its pressure regulation, transport dynamics, and simulated/computational modelling, as the foundation for next-generation drug delivery design. While diagnostic and sampling technologies are summarized for context, the review primarily explores how ISF dynamics guide targeted therapy and precision medicine. Overall, ISF offers a dynamic and responsive medium for next-generation medical applications, combining real-time physiological insight with localized therapeutic action. Furthermore, current research and technological advancements, emphasizing the transformative potential of ISF to bridge diagnostics, personalized therapy, as well as drug development and targeted delivery are reviewed herein.

**Graphical abstract:**

**Figure:**
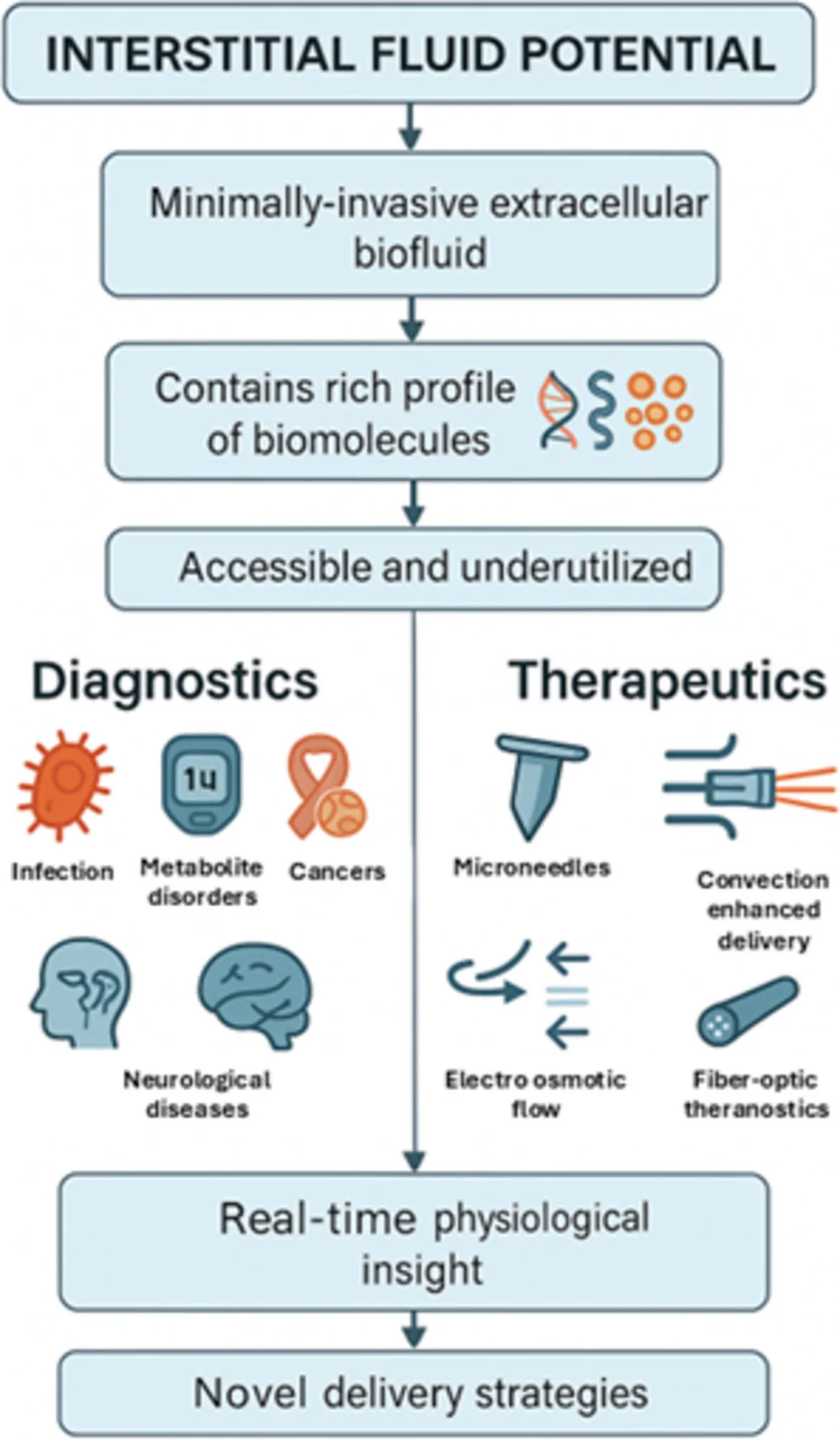

## Introduction

**Fig. 1:**
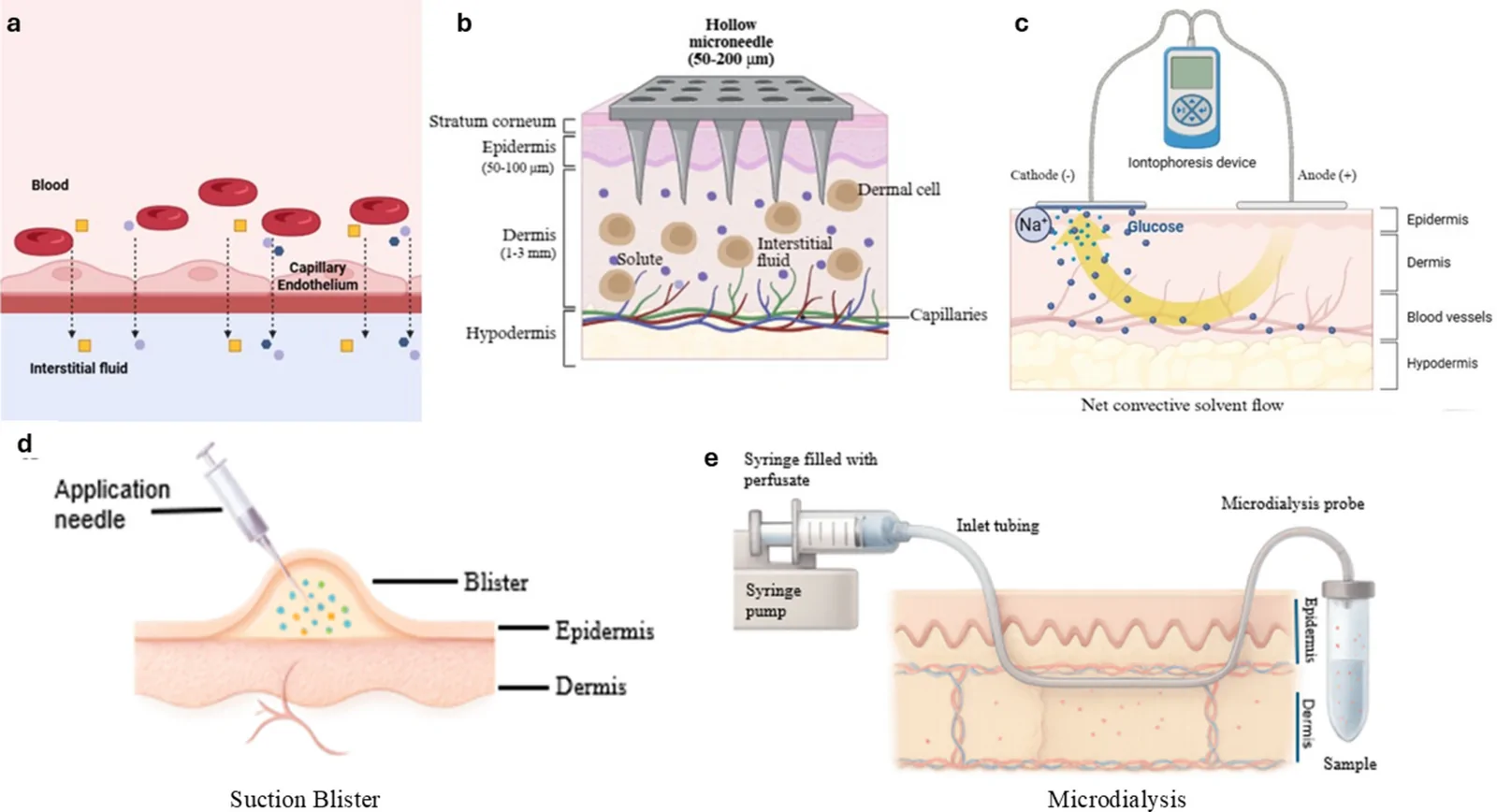
Schematic of interstitial fluid (ISF) extraction and biomarker transport dynamics in skin tissue. (**a**) Transport of biomarkers from blood to ISF across capillary endothelial barriers. (**b**) MN-based approach to access ISF in the dermal layer, where capillaries are abundant and ISF is in close proximity to systemic circulation, MNs penetrate the skin into the superficial dermis, enabling minimally invasive, low-volume ISF extraction compatible with wearable and continuous sensing platforms. (**c**) Iontophoretic extraction showing directional migration of neutral solutes such as glucose alongside charged solutes such as Na⁺ from dermal tissue toward the cathode under an applied electric field, facilitating ISF sampling via electroosmosis. (**d**) Suction blister, where negative pressure separates the epidermis from the dermis, enabling collection of ISF from superficial dermal layers. (**e**) Microdialysis, in which a semipermeable probe is implanted into the dermis or subcutaneous tissue to continuously sample ISF with temporal resolution of minutes. (Created using images from Biorender.com and Illustrae)

Interstitial fluid (ISF) is the extracellular fluid that nourishes cells and maintains tissue homeostasis by facilitating the exchange of nutrients, gases, and waste products between blood capillaries and cells [1]. It regulates osmotic balance, pH, and metabolic waste removal, ensuring optimal cellular function and tissue health [2]. ISF constitutes 75% of extracellular fluid, representing 15–25% of total body weight [3]. The skin (the body’s most accessible organ) contains the highest density of extractable ISF in the dermis, its vascularized middle layer [4]. Here, ISF is continuously generated via transcapillary filtration where blood plasma, and its contents, move through the capillary walls and into the interstitial space [5]. Importantly, ISF has a rich and diverse biomarker and analyte composition which is similar to that of blood [6], a relationship that arises from continuous exchange across capillary endothelial barriers. As illustrated by Fig. 1a, small molecules (< 3 kDa), including ions, glucose, and urea (denoted by blue circles in illustration), readily diffuse across capillary walls and therefore display ISF concentrations comparable to blood plasma [7]. For intermediate-sized biomarkers (3–70 kDa), such as insulin, cytokines, and albumin (shown as purple circles in illustration), transport is partially restricted by size and governed by coordinated paracellular and transcellular mechanisms. In contrast, macromolecules exceeding 70 kDa (indicated by yellow squares in illustration) may exhibit reduced ISF concentrations due to limited permeability across endothelial barriers [7].

In the skin, dermal ISF is located beneath the epidermis, typically starting at a depth of roughly > 100 µm and in close proximity to dense capillary networks, making it accessible using minimally invasive sampling strategies, such as microneedle (MN)-based approaches (Fig. 1b). These kinds of minimally invasive sampling strategies enable direct access to ISF within the dermal interstitium without the need for venepuncture [8]. As illustrated in Fig. 1c, iontophoresis further facilitates ISF extraction by driving both charged species (e.g., Na⁺) and neutral analytes (e.g., glucose) toward the electrode via electroosmotic flow. Owing to its localized interactions with surrounding cells, certain biomarkers may appear earlier or at higher relevance in ISF compared with blood, which reflects a pooled signal from multiple organs [9]. A quantitative comparison demonstrated that dermal ISF closely reflects the biomolecular composition of blood while exhibiting systematic differences in concentration and partitioning. Protein analysis showed that although the relative composition of major serum proteins was preserved in dermal ISF, their absolute concentrations were lower. Total protein averaged 40.9 g/L in dermal ISF compared to 70.9 g/L in serum, corresponding to a serum-to-dermal ISF ratio of 1.73 [10]. Similar trends were observed for albumin and IgG, with ratios of 1.89 and 2.15, respectively, while the proportional distribution of these proteins remained comparable between matrices. Overall, dermal ISF contained approximately 50–60% of the protein levels found in serum, supporting the concept that dermal ISF represents an ultrafiltrate of plasma [10]. Analysis of inflammatory and cardiovascular biomarkers further confirmed the presence of diagnostically relevant analytes in dermal ISF, although concentrations were generally diluted relative to serum and varied according to molecular size. Several cytokines showed significant correlations between the two matrices, while some biomarkers were enriched in dermal ISF, highlighting its ability to capture both systemic and local tissue-derived signals. Together, these observations reinforce the biochemical similarity between blood and ISF while illustrating the unique analytical value of the interstitial compartment. Another advantage afforded by ISF is that it does not clot, which makes its handling a lot more straightforward, and the continuous monitoring of its biomarkers using wearable devices becomes more feasible [11]. These anatomical and transport features (summarized in Fig. 1a-c) position ISF as a promising candidate for diagnostic monitoring and therapeutic research, particularly in the development of minimally invasive sensing and drug delivery platforms. Furthermore, the advances made in the use of ISF may have exciting outcomes in the field of precision and personalized medicine [7, 12].

### Emerging harvesting technologies for ISF: anatomical sources and biomarker signatures

There is rapidly increasing interest in personalized medicine and point of care diagnostics using novel biomarkers. Biofluid sampling is the first essential step for biomarker associated in vivo metabolic analysis [13]. A biofluid which is considered to be an ideal candidate for metabolic analysis is sweat, which is characterized as a noninvasively collectable biofluid [14]. Sweat hosts a broad range of biomarkers and is available on almost all parts of the human body [15]. In contrast, blood remains the primary source of body fluid containing these biomarkers [16], due to its complex and well-characterized composition [17]. However, blood sampling by venipuncture may cause discomfort for some patients and requires trained medical professionals, while capillary blood sampling is considered less invasive [16]. Alternate body fluids such as urine and saliva are more easily accessible but have their own limitations. Urine testing poses privacy and adulteration concerns, increasing interest in oral fluid as a less invasive, more secure alternative. Oral fluid allows observed collection and resists tampering but has a shorter drug detection window (30 min to 24 h) [18]. Drug levels in oral fluid depend on pH, with basic drugs like amphetamines showing higher concentrations. Unlike urine, which contains metabolites, oral fluid reflects parent compounds, limiting some immunoassay effectiveness [18]. Standardization remains limited, though recent Substance Abuse and Mental Health Services Administration (SAMHSA) guidelines have introduced consistent cutoff values [18]. Despite these concerns, the potential for ISF as an alternative source extraction of biomarkers has gained the attention of researchers.

The successful extraction of ISF which has recently emerged as a valuable biofluid for physiological and pathological changes within specific tissues [16], depends greatly on both the anatomical location of harvest and the technique employed, as these factors directly influence the quality and relevance of the fluid obtained [19]. The anatomical proximity of the fluid to certain cellular environments reveals tissue-specific microenvironmental signatures, which cannot necessarily be predicted using other fluids [20]. Therefore, ISF harvesting can thus be strategically aligned with disease-specific regions, which allows the enhancement of diagnostic precision [21]. Furthermore, since a more critical insight is provided into the biochemical environments, this allows for a more targeted and effective drug delivery strategy.

### Methods of ISF extraction: from conventional approaches to wearable technologies

A commonly used approach to collect dermal ISF is the suction blister technique, which involves applying negative pressure, generally kept between −7 inHg and −11 inHg throughout the procedure, to the skin to separate the epidermis from the dermis, followed by aspiration of the accumulated blister fluid using a hypodermic needle and syringe (Fig. 1d) [22]. This method typically accesses superficial dermal ISF at depths of ~ 100–300 µm and yields relatively large sample volumes (16.6–156 µL per blister) [22]. However, the prolonged suction (often 1–3 h) induces local tissue stress and inflammation, and ISF collected using this method has been shown to contain biomarkers associated with tissue injury, such as cytokines and damage-associated molecular patterns, which may compromise its physiological relevance [3]. These limitations, together with the time-consuming nature of blister formation, have restricted its widespread clinical adoption [19]. At present, increasing biofluid sampling methods have been explored, such as microdialysis, ultrafiltration, needle-based extraction and wearable sensors. Among them, needle-based extraction has been recognized as the most common and reliable method in clinical settings [23].

Microdialysis is a well-established technique for sampling extracellular molecules from ISF, particularly in neuroscience and metabolic research (Fig. 1e). In this approach, a microdialysis probe containing a semipermeable membrane with high-molecular weight cut off membranes (100 kDa-3 MDa) is implanted into the dermis or subcutaneous tissue at depths ranging from 1–10 mm [24]. ISF analytes diffuse across the membrane into a continuously perfused carrier fluid and are collected as dialysate with high temporal resolution (sampling intervals of 1–30 min) [25]. However, analyte recovery is incomplete and highly dependent on perfusion rate, membrane properties, and molecular size, with typical relative recoveries ranging from 10–40% for small metabolites [26]. While microdialysis enables dynamic monitoring of ISF composition, its invasiveness, need for external pumps, and low absolute sample volumes limit its suitability for routine or wearable applications.

Traditionally, ultrafiltration has been used to separate chemicals, but recently this minimally invasive technique is applied to sample tissue ISF by implanting capillary ultrafiltration probes that apply negative pressure to draw ISF across a semipermeable membrane (molecular weight cut-off > 30 kDa), with reported membranes with molecular weight cut-off of 400 kDa being utilised to afford collection of proteins in tumor interstitial fluid (TIF) [27]. The method is designed to mimic physiological fluid movement, allowing passive filtration of ISF without causing significant tissue damage. This technique samples ISF at similar depths to microdialysis (millimetre range) and can yield higher absolute analyte recovery, as fluid rather than solutes alone is collected. However, prolonged application of negative pressure may perturb local fluid balance, and probe implantation remains invasive, limiting broader clinical translation [28].

In recent years MNs have emerged as a promising minimally invasive approach for ISF extraction (Fig. 1b). MNs consist of protrusions with sharp tips (typically > 300 µm in length) that penetrate the skin and access ISF within the superficial dermis without reaching nerve endings [23, 29]. MN-based sampling typically yields nanolitre to microlitre-scale ISF volumes and offers superior patient comfort and compatibility with repeated or continuous sampling. Studies using 3D-printed MN arrays and hollow or swellable MN designs have demonstrated successful extraction of ISF biomarkers, including glucose, lactate, and electrolytes, with the fastest record collecting an average of 15.5 mg of ISF in 5 min utilizing 154 puncture sites in humans [30]. Importantly, MNs are readily integrated with wearable sensors, enabling near real-time monitoring of ISF biomarkers.

Lastly, wearable sensors have garnered interest in biomedicine to continuously measure and screen biomarkers in the human body. Current ISF sensors mainly rely on implanted probes and has been investigated on a limited number of biomarkers such as glucose [31]. This technique is still in the pilot stages, and researchers are trying to find a solution to make wearable ISF biosensors less invasive and more comprehensive. Recently, researchers have presented a wearable MN-based sensor for continuous and real time sensing of metabolites found in ISF [32]. This technology exemplifies significant advancements in the development of MN sensing platforms for personalized healthcare.

Table 1 provides further research summarising the advances in ISF sampling across various anatomical sites, accentuating the diversity of harvesting techniques, biomarker types, and key findings. Each method demonstrates unique advantages in terms of ISF accessibility, biomarker yield, and clinical relevance. For instance, dermal ISF obtained via MN-based platforms shows strong overlap with plasma biomarkers, supporting its potential as a non-invasive diagnostic alternative. Similarly, gingival crevicular fluid (GCF) and tumor-derived ISF have yielded clinically meaningful cytokine profiles. Emerging techniques, such as micro-invasive neural probes and volume-metered devices, offer enhanced spatial precision and sampling accuracy, broadening the utility of ISF in biomedical research.

**Table 1:** Anatomical sources of interstitial fluid (ISF): Harvesting techniques, biomarkers, and key findings

ISF Source	Harvesting Technique(s)	Key Biomarkers (Examples)	Key Findings
Skin (Dermal ISF)	MN Patches [11] MN POP Device [30] MN Mechanical pressure/microfluidic chip device [33]	Cytidine and CMP (cancer biomarkers), 3-methyladenine (biomarker of DNA damage) and inosine (cardiac biomarker and neuroprotective agent for possible treatment of Parkinson’s disease and multiple sclerosis) [11] Proteins [30] Caffeine (test compound) [33]	Most biomarkers detected in plasma are also in ISF, confirming ISF as an alternative substitute for blood for diagnostic tests [11] 149 of the identified ISF proteins are in the National Cancer Institute Early Detection Research Network Biomarker database [30] Preliminary findings indicate that the developed device enables reliable, volume-metered sampling of ISF, delivering 1.1 µL ± 3.5% [33]
Oral Cavity (Gingival Crevicular Fluid—GCF)	Silk ligature [34]	Cytokines [34]	A novel GCF sampling method has proven effective for cytokine measurement, enabling the detection of dynamic changes associated with periodontal bone loss in a mouse model of periodontal disease [34]
Central Nervous System	Micro-invasive platform [35] Microdialysis [36]	Neural proteins [35, 36]	Micro-invasive probes offer a significantly smaller spatial footprint compared to conventional sampling devices, enabling higher spatial resolution and minimizing inflammation in surrounding tissues [35] Identified and better detected new potential biomarkers for Alzheimer’s disease [36]
Tumors (Tumor Interstitial Fluid—TIF)	Adsorbent paper strips [37] Centrifugation [38]	Cytokines IL-1β [37] TIF proteins [38]	Results demonstrated that absorbent paper strips effectively collected breast TIF in sufficient quantities for IL-1β analysis in invasive ductal carcinoma samples [37] Insight into tumor growth and metastasis is provided, through proteomic analysis of TIF [38]

Key: MN = Microneedle, POP = Puncture-Out-and-Press

A quantitative comparison of ISF sampling approaches highlights clear trade-offs between sampling depth, temporal resolution, sample volume, and invasiveness. Suction blister techniques typically yield relatively large volumes of ISF (approximately 16–156 µL per blister) but require prolonged sampling times (1–3 h) and can induce localized inflammation. Microdialysis enables continuous sampling with temporal resolution on the order of minutes; however, analyte recovery is often limited, typically ranging from 10–40% depending on molecular size and perfusion rate. Ultrafiltration probes allow bulk fluid collection and consequently higher analyte recovery, though probe implantation remains invasive. In contrast, microneedle-based platforms generally collect smaller volumes (nanolitre to low microlitre scale) but enable rapid sampling (seconds to minutes) with minimal tissue disruption and improved compatibility with wearable biosensing systems. These quantitative differences illustrate that no single technique is universally optimal; rather, method selection should be guided by analytical requirements, target biomolecules, and clinical context. Collectively, these approaches also illustrate a clear technological shift from high-yield but invasive techniques toward minimally invasive, scalable platforms suitable for longitudinal monitoring. Rather than maximizing sample volume alone, emerging strategies increasingly prioritize physiological fidelity, patient compliance, and real-time feedback relevant to therapeutic monitoring and dose optimization. Hybrid systems integrating microneedles with microfluidics, biosensors, and controlled-release platforms represent a particularly promising direction, aligning ISF extraction modalities with clinical context, therapeutic intent, and system-level integration.

### Quality of ISF harvesting: anatomical sites, and their diagnostic and clinical relevance

Recognizing ISF as a tissue-proximal and diagnostically rich biofluid naturally shifts attention toward how and where it is harvested, since anatomical location and sampling strategy critically determine biomarker fidelity, as discussed above. This has driven increasing focus on evaluating the quality, accessibility, and clinical relevance of ISF obtained from distinct anatomical compartments, where site-specific microenvironments impart unique biochemical signatures with direct implications for diagnosis, monitoring, and targeted therapeutic intervention. In this context, the hypodermis (subcutaneous tissue) [19], located just beneath the dermis [39], is also a highly favored practical site for harvesting ISF due to its relative ease of access, minimal invasiveness, and suitability for continuous monitoring [11]. The quality of ISF obtained from subcutaneous tissue is generally high in terms of sample purity and biomarker relevance, particularly for applications in metabolic, endocrine, and inflammatory disease monitoring. ISF in subcutaneous tissue exhibits a strong correlation with plasma concentrations of key biomarkers such as glucose, lactate, and cytokines, making it a reliable alternative to blood sampling for real time monitoring [40].

**Fig. 2:**
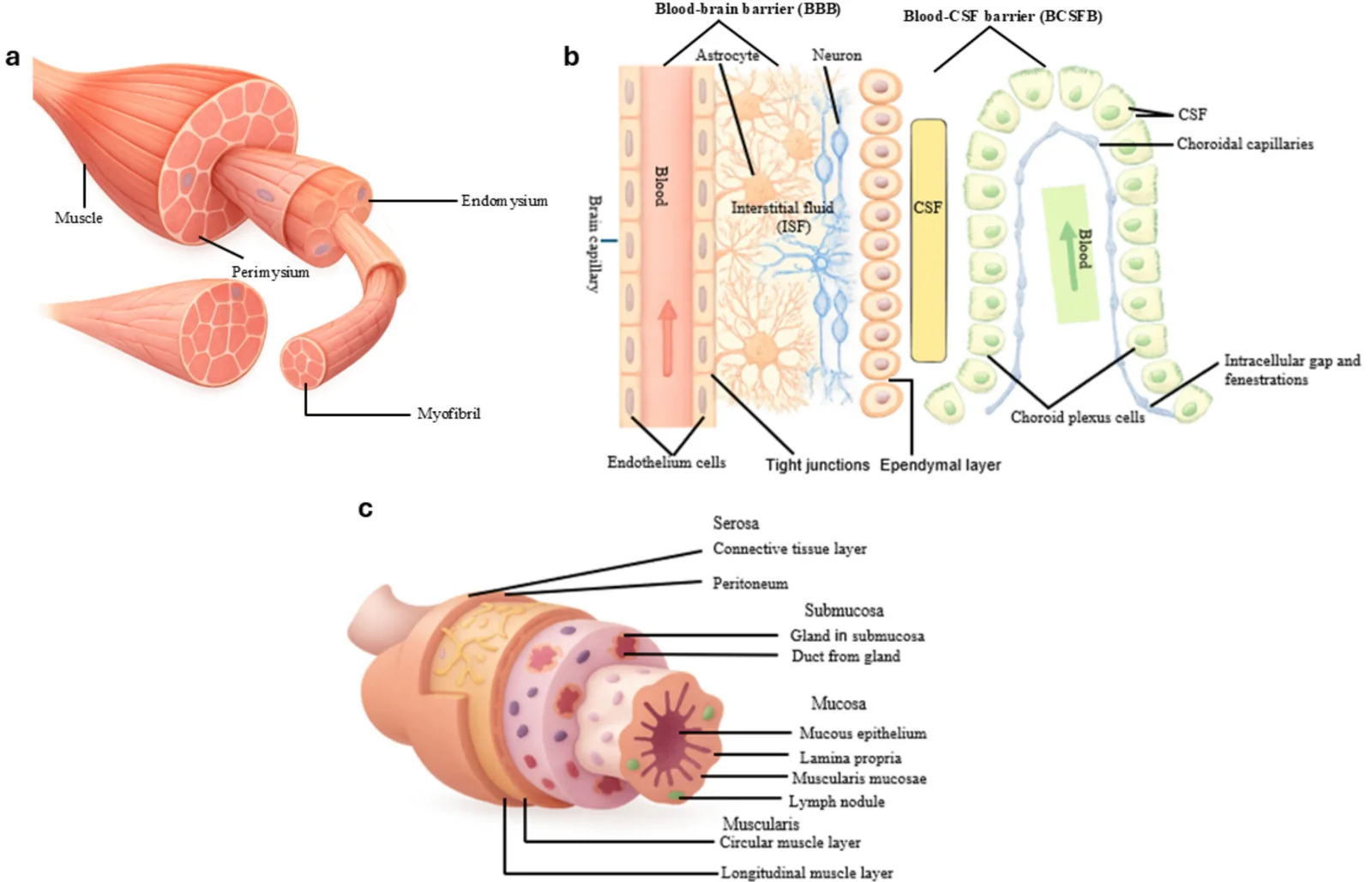
Anatomical sites relevant for ISF sampling, representing key tissue compartments where ISF may be accessed for diagnostic or therapeutic purposes. (**a**) Schematic of skeletal muscle tissue showing hierarchical structure from whole muscle to individual myofibrils. ISF resides between muscle fibers, within the endomysium and perimysium, facilitating molecular exchange and signalling. (**b**) Illustration of how the blood brain barrier (BBB) and blood-cerebrospinal fluid barrier (BCSFB) regulate solute exchange between blood, ISF, and CSF. The BBB involves endothelial tight junctions and astrocytes. The BCSFB is formed by choroid plexus epithelial cells connected by tight junctions, while the underlying choroid plexus capillary endothelium is fenestrated. (**c**) Cross-sectional anatomy of the GIT depicting the multi-layers of mucosa, submucosa, muscularis, and serosa. The submucosal space and lamina propria are key ISF reservoirs supporting nutrient exchange, immune surveillance, and signalling. (Created with images from Illustrae)

The extraction of ISF from muscle tissue provides a direct window into the metabolic and physiological status of skeletal muscle, offering valuable diagnostic and monitoring potential for both metabolic disorders and muscular diseases. Each muscle fiber (approximately 100 µm in diameter and 1 cm in length) is ensheathed by a fine reticular connective tissue layer forming the endomysium. Together with the perimysium and epimysium, these connective tissue compartments provide the structural framework for vascularization and innervation, thereby facilitating efficient exchange between muscle fibers, the surrounding interstitial space, and the microcirculation, as illustrated in Fig. 2a [41, 42]. Investigations by Zhang et al. pioneered a study using minimally invasive catheter-based sampling of ISF from rat gastrocnemius muscle, as well as microdialysis sampling in human vastus lateralis muscle. Their non-targeted metabolomics analysis revealed 299 and 414 unique metabolites in rat and human muscle ISF, respectively. Notably, only 43% of the metabolites were shared between muscle ISF and blood, highlighting the tissue-specific nature of ISF composition. Exercise was found to induce substantial shifts in ISF metabolite profiles, including increased levels of amino acids (e.g., aspartate, methionine), tricarboxylic acid (TCA) cycle intermediates (e.g., citrate, α-ketoglutarate), and signalling lipids such as oleamide. Furthermore, several metabolites like lactate and β-hydroxybutyrate increased in response to exercise, aligning with expected metabolic fuel shifts. In humans, approximately 70% of the exercise-responsive metabolites observed in rats were similarly altered, supporting the translational relevance of ISF profiling [43]. This study demonstrated that muscle ISF metabolomics is a powerful, minimally invasive approach to assess muscle-specific metabolic changes in real time and could serve as a valuable tool in both research and clinical settings. In the context of metabolic disorders such as type 2 diabetes and insulin resistance, muscle ISF analysis has been instrumental in revealing impaired glucose uptake and altered energy substrate utilization, which are hallmarks of disease progression [44]. Similarly, in muscular dystrophies and myopathies, ISF biomarkers such as creatine kinase, cytokines, and reactive oxygen species have been detected at elevated levels, reflecting localized muscle injury and inflammation [45]. This localized fluid analysis allows for a more refined understanding of disease mechanisms than blood-based biomarkers.

The cerebrospinal fluid (CSF), which surrounds the brain and spinal cord reflects the biochemical environment of the CNS more directly than blood or peripheral fluids [46]. The CSF provides another example of how anatomical barriers shape ISF composition and diagnostic value. As depicted in Fig. 2b, the biochemical composition of CNS ISF and CSF is tightly regulated by the blood–brain barrier (BBB) and the blood-cerebrospinal fluid barrier (BCSFB), which restrict passive diffusion from blood and confer high anatomical and molecular specificity to CNS-derived biomarkers. The BBB is formed by specialized capillary endothelial cells lining the brain parenchyma, separating blood from CNS interstitial fluid and severely limiting the entry of hydrophilic, high molecular weight, or neuroactive agents. These endothelial cells, together with astrocytes, pericytes, neurons, and the basement membrane, constitute the neurovascular unit. In contrast, the BCSFB is located in the choroid plexus of the cerebral ventricles and consists of a highly vascularized stromal core lined by epithelial cells. Functionally distinct from the BBB, the BCSFB is comparatively more permissive, containing pores and vesicular transport pathways that allow selected water-soluble molecules to enter the CSF in a molecular weight-dependent manner [47]. The BBB and BCSFB facilitate solute exchange between blood, ISF, and CSF through endothelial tight junctions, astrocytic end-feet, and specialized choroid plexus epithelium. These barriers confer high specificity to CNS-derived ISF biomarkers, making it a more sensitive and informative fluid than blood for neurological disorders. The quality of ISF-derived biomarkers from CSF is superior in terms of specificity and sensitivity for neurological conditions, as it is in close proximity to the site of pathology in neurodegenerative, inflammatory, and neoplastic CNS disease [48]. The CSF can act as a reliable source of biomarkers for the diagnosis and treatment of monitoring different neurological diseases, including neurodegenerative diseases such as Alzheimer’s, Parkinsons as well as primary and secondary brain malignancies. Incorporating these CSF biomarkers into drug discovery and development allows for a more efficient diagnosis with a higher success rate [49].

Despite ISF being a rich source of biomarkers, a drawback is poor accessibility. Considering this, the skin has proved to be a relatively easier route to extract available biomarkers. However, extracting ISF from skin can be challenging as skin is not homogenous, and varies anatomically depending on the location [50]. Sampling skin ISF is an emerging strategy being explored for clinical diagnostics and biomarker monitoring, particularly in the development of minimally invasive technologies [51], as biomarkers extracted from skin ISF may be more valuable for diagnostic or patient monitoring purposes, especially for localized diseases. Skin ISF can be used for continuous glucose monitoring [50]. Building on this, a recent study developed a tilted microneedle interstitial fluid collecting system (TMICS), fabricated using 3D printing, that significantly enhances ISF extraction efficiency by optimizing MN insertion angle and force. The system enabled rapid (within 30 s) and reproducible ISF collection (up to 2.9 μL in vivo), offering a promising route for high-speed, minimally invasive sampling suitable for multiplexed biomarker detection applications [16].

Researchers have also investigated ISF extracted from the Gastrointestinal tract (GIT), which is the interface between the host and its microbiota. It is assumed that the ISF harvested from the intestinal tissue is rich in both host produced and microbially produced factors such as cytokines, metabolites, and proteins [52]. As shown in Fig. 2c, the submucosa and lamina propria beneath the intestinal epithelium serve as key ISF reservoirs supporting immune surveillance, nutrient exchange, and host-microbial signalling. The lamina propria is rich in immune cells, capillaries, and lymphatics, making it a primary ISF-rich site for immune surveillance and signalling [53]. The submucosa contains larger blood and lymphatic vessels and contributes to fluid exchange and transport [54]. Both layers are central to nutrient absorption, cytokine diffusion, and microbiota-host communication. Avery et al. developed and validated centrifugation and elution-based methods to isolate intestinal ISF from various segments of the GIT, **(**Fig. 2c**)**, in both rodents and humans, enabling site-specific profiling of the mucosal microenvironment. Their study revealed significant differences in the molecular composition of ISF across intestinal segments, with localized enrichment of antimicrobial peptides (e.g., REG3G, DEFA), cytokines (e.g., IL-18, IL-1β), and microbial metabolites such as short-chain fatty acids. Proteomic analysis showed that intestinal ISF has a distinct protein signature compared to serum, underscoring the value of ISF for accessing local biochemical activity that cannot be inferred from systemic sampling alone. Notably, the elution-based technique was successfully translated to human colonoscopic biopsies, revealing disease-specific cytokine profiles in patients with inflammatory bowel disease (IBD), thus highlighting the clinical utility of ISF in personalized diagnostics and therapeutic monitoring [55].

Taken together, these findings indicate that the diagnostic and translational value of ISF is not intrinsic to the fluid itself but is critically determined by anatomical context, barrier architecture, and sampling feasibility. Subcutaneous and skin ISF offer high accessibility and temporal resolution but primarily reflect systemic or superficial processes, making them well suited for continuous monitoring rather than deep tissue specificity. In contrast, muscle, CNS, and gastrointestinal ISF provide markedly higher biochemical resolution and disease relevance due to proximity to pathological processes, albeit at the cost of increased invasiveness and technical complexity. This creates a fundamental translational trade-off between sampling ease and biomarker specificity. From a drug delivery perspective, these compartmental differences also dictate transport dynamics, residence time, and therapeutic targeting potential. Importantly, emerging technologies such as microneedle arrays, catheter-based microdialysis, and site-adapted ISF extraction platforms, which will be discussed in later sections, are beginning to shift this balance by improving access to high-value ISF compartments. Thus, rational selection of ISF harvesting sites should be guided by disease localization, required analytical depth, and delivery objectives rather than convenience alone, positioning ISF as a strategically deployable biofluid in precision medicine.

It is important to note that ISF originates from transcapillary filtration and is cleared into the venous circulation by lymphatic vessels [56]. Its composition is dependent on substances that are either produced and secreted locally, or by substances that are transported to the tissue by circulation [57]. Under normal circumstances, the volume of ISF is kept constant by several homeostatic structural processes, such as force adjustment across the capillary wall, as well as lymph flow [58]. The composition and biophysical properties of ISF, however, do vary in instances where there is pathogenesis, inflammation, remodelling, as well as during normal functional cycles [58–60]. Building directly on the homeostatic regulation and context-dependent variability of ISF, this regulated transcapillary filtration and lymphatic clearance also underpin how ISF redistributes across anatomical compartments. In organs located in the pleural, pericardial, and peritoneal spaces, some ISF filters through the organ's serosal surface into the surrounding fluid space and then taken up into the lymphatic system [61, 62]. In the oedematous intestine, ISF can cross the mucosal barrier into the lumen [54]. In the skin, ISF is found in the dermis. Approximately 70% of this layer of the skin is made of water with 40% of this water constituting ISF [63]. The dermis interacts with the systemic blood circulation, facilitating fluid movement through capillaries into the interstitial space [11]. ISF is therefore derived from blood surrounding tissues and cells, playing a crucial role in transporting nutrients and signalling molecules [64]. The transfer of biomarkers or analytes between blood and ISF occurs primarily due to hydrostatic and osmotic pressure gradients at the dermal-blood interface. Consequently, ISF composition at the dermis closely resembles that of blood and provides localized physiological insights [50].

As a direct consequence of this close physiological and compositional coupling between dermal ISF and the systemic circulation, skin ISF represents a uniquely information-rich biofluid for molecular profiling. This is exemplified by metabolomic and proteomic analyses of microneedle-sampled skin ISF, which have revealed a diverse array of clinically relevant biomarkers. This was demonstrated by a metabolome study of skin ISF sampled via microneedling by Samant et al*.,* which revealed novel information on clinically relevant biomarkers that were found in skin ISF [11]. Briefly, this study found that nucleosides such as cytidine and CMP (cancer biomarkers), 3-methyladenine (biomarker of DNA damage) and inosine (cardiac biomarker and neuroprotective agent for possible treatment of Parkinson’s disease and multiple sclerosis) are mostly found in the skin ISF, but are absent in the blood [11]. Proteins that are found in skin ISF include cytokines (e.g., TNF-α, IL-6) and growth factors (e.g., VEGF) and are key indicators of inflammatory and neoplastic conditions [65]. Enzymes such as matrix metalloproteinases (MMPs) are also found in the skin ISF and they are implicated in tissue remodelling and metastatic progression [66, 67]. Antibodies in ISF, including IgG and IgE, reflect immune responses in allergies and autoimmune disorders [68, 69]. Signalling molecules such as prostaglandins and neuropeptides modulate pain and inflammation in chronic diseases like psoriasis and neuropathy [70, 71]. Metabolites like glucose and lactate serve as dynamic biomarkers for diabetes and hypoxia-related conditions [72, 73]. Circulating cell-free DNA (cfDNA) which is typically released into blood after programmed cell death and necrosis may also be detected and continuously monitored in ISF [74]. The concentration, integrity, genetic, and epigenetic alternations in this cfDNA may suggest pathological conditions of the body, such as inflammation, autoimmune diseases, stress, or even cancer [75].

Surprisingly, despite this rich biomolecular content and growing evidence of its diagnostic value, the application of ISF in the context of infectious diseases remains remarkably underexplored. Given that ISF contains key immunological and inflammatory mediators such as cytokines, antibodies, and cfDNA, that are central to host–pathogen interactions, it holds untapped potential for use in the detection, monitoring, and possibly even prognosis of infectious diseases. Future research should consider leveraging these established ISF components, which are originally studied in oncology, neurology, and metabolic diseases, for the identification of infection-related biomarkers, real-time immune monitoring, and understanding tissue-specific immune responses during infection.

The use of microneedling to sample and detect several microRNAs (miRNAs) has also been explored, and these microRNAs show potential as biomarkers for the early detection of a wide range of diseases [76–78]. It has also been found that one of the barriers to successful cancer treatment is an elevated interstitial fluid pressure (IFP) caused by inadequate tumor perfusion from absent lymphatic vessels, a leaky and immature tumor vasculature, an overly stimulated angiogenesis, and from interstitial fibrosis [79]. When IFP is elevated, convection-driven fluid transport within tumors is hampered, causing inefficient penetration and distribution of anti-neoplastic drugs [80, 81]. Strategies to overcome or treat high IFP are therefore being investigated. These strategies include, vascular normalization, which aims to restore the structure and function of tumor vasculature. This is one of the proposed strategies for overcoming the high IFP in tumors to make drug delivery more effective [82]. The use of anti-VEGF receptor antibodies and tyrosine kinase inhibitors to inhibit angiogenesis in the tumor microenvironment is one of the therapeutic interventions that has showed promise in normalizing vasculature and thus reducing IFP [83–85]. When it comes to some novel strategies, Fu et al*.* developed a photoactivated Z-scheme nanomotor with spatiotemporally synchronized O_2_ self-supply and ROS generation capability under NIR laser irradiation for reducing the high IFP in tumors to enhance deep tumor delivery of drugs and hypoxic tumor therapy [86].

### Current research in ISF biomedical applications

ISF has shown promise as a valuable tool for the early detection of infections as it harbours infection-related biomarkers such as bacterial metabolites, inflammatory proteins, as well as certain nucleic acids [87, 88]. For instance, pyocyanin, a metabolite produced by certain bacteria, can be directly detected in ISF and serves as a specific biomarker for bacterial infections [87]. Sensitive MN-based sensors have demonstrated reliable detection of pyocyanin in animal models and wound sites, supporting its use for early infection diagnosis [87]. Inflammatory cytokines and pathogen-specific proteins (e.g., *Plasmodium falciparum* histidine-rich protein 2 for malaria) are detectable in ISF using MN patches and lateral flow assays [89–91]. These proteins can indicate the presence of infection and the body’s immune response.

Cell-free nucleic acids, such as microRNAs and pathogen-derived DNA/RNA, are present in ISF and can be sampled using specialized MN arrays. These nucleic acids provide information about infection status and disease progression [90, 92]. Current research in ISF biomarker discovery is rapidly advancing, with a strong focus on minimally invasive sampling and real-time monitoring technologies. Multiple studies highlight the development of MN patches and arrays for extracting ISF and detecting biomarkers such as proteins, nucleic acids, metabolites, and microRNAs. These devices are designed for minimal discomfort, high sampling efficiency, and rapid analysis, making them suitable for point-of-care and continuous monitoring applications [7, 19, 93]. Recent innovations include encoded MN arrays with photonic crystal barcodes for multiplexed detection, hydrogel-coated MNs for nucleic acid capture, and colorimetric sensors for visual biomarker readout. These platforms allow simultaneous detection of multiple biomarkers and can be integrated with smartphone apps for real-time data visualization [94–96].

From a transport perspective, ISF composition is governed by the interplay between capillary filtration, extracellular matrix permeability, and lymphatic drainage. These parameters collectively regulate IFP, solute diffusion, and convective flow. Consequently, any technology designed to sample or deliver therapeutics through ISF must operate within these physical constraints. Sampling methods therefore differ not only in invasiveness but also in the extent to which they perturb local interstitial pressure gradients or disrupt extracellular matrix structure. Establishing this mechanistic framework is essential for interpreting biomarker measurements and designing ISF-targeted drug delivery systems.

Considering these advancements, this review prioritizes the biophysical role of ISF, specifically fluid transport, ECM/IFP regulation, and the use of simulated and computational ISF models, to guide next-generation drug delivery system design. Despite increasing interest in ISF diagnostics, relatively few reviews have examined how the physical transport properties of ISF directly influence therapeutic delivery, creating a gap that this review aims to address. Further focus herein is to comprehensively assimilate current knowledge on the composition and potential clinical significance of ISF biomarkers, while critically evaluating the latest technologies enabling their detection. It also provides a concise incursion on how these innovations intersect with the broader landscape of precision and personalised medicine, emphasizing the potential of ISF as a tool for real-time diagnostics, individualized disease monitoring, and targeted drug delivery. By integrating insights from biofluid dynamics, molecular biology, and biomedical engineering, we provide a critical appraisal of the translational relevance of ISF in overcoming current limitations in healthcare, particularly in achieving more personalized, predictive, and responsive therapeutic strategies.

## Fluid dynamics and transport

The ISF compartment, which comprises the fluid and extracellular matrix (ECM) surrounding cells, is a critical nexus for physiological exchange, mediating the transport of nutrients, waste, and therapeutic agents between the vasculature and the parenchyma [61]. Collagen fibres and glycosaminoglycans such as hyaluronan form a mechanical scaffold that influences ISF viscosity, flow resistance, and molecular diffusion [97, 98]. Physiological stimuli, including exercise or local metabolic activity, can transiently alter these forces, affecting interstitial volume and solute transport [99]. When this balance is disrupted, such as through increased capillary permeability or reduced oncotic pressure, pathological fluid accumulation and edema may occur [100]. The dynamics of ISF, which specifically include its pressure, velocity, and composition, are profoundly altered in pathological states. This is most notable in solid tumors where the microenvironment is transformed into a formidable barrier for drug delivery and provides novel avenues for non-invasive methods of diagnosis [101–103]. A critical evaluation of recent literature reveals that quantitative modelling and the recognition of compartmental differences in ISF dynamics are essential for advancing both personalized medicine and the design of next-generation drug delivery systems.

### Quantitative models of ISF dynamics

The foundation for understanding ISF dynamics rests on classical fluid mechanics principles, which have been adapted to model the complex, porous nature of the tissue interstitium. The exchange of fluid between the microvasculature and the interstitium is classically described by the Starling Principle, which balances hydrostatic and colloid osmotic (or oncotic) pressure gradients to transport fluid out of and into the capillaries, respectively [104]. The traditional model has however, been refined to the Revised Starling Principle, which recognizes the endothelial glycocalyx layer as the primary semipermeable barrier [105]. This revised principle suggests that net fluid absorption is transient in most tissues, with a slight net filtration prevailing in the steady state [106]. Furthermore, this principle further shows that damage to the glycocalyx (e.g., in inflammation or sepsis) significantly alters fluid balance, leading to massive oedema that can affect drug distribution pathways significantly [107].

Fluid movement through tissues is governed by the pressure gradient across the porous ECM, which is quantitatively described by Darcy's Law [108]. This law relates interstitial fluid velocity (IFV) to hydraulic conductivity as well as pressure gradient and is written as follows: u =  − K∇p, where u is the IFV, K (mm^2^/s/mm Hg) is the hydraulic conductivity of the interstitium, and p (mm Hg) is the IFP [109, 110]. Importantly, the hydraulic conductivity is a parameter of the tissue which characterizes the ease with which a fluid moves through a porous media [111].

### Compartmental differences and physiological impact

IFP is a critical determinant of solute transport and drug distribution within tissues. In healthy tissue, IFP typically ranges from –1 to + 2 mmHg, allowing outward plasma filtration that is balanced by lymphatic clearance [112]. In tumours, fibrosis, and chronic inflammation, IFP can rise above + 10 mm Hg due to vascular leakage, ECM remodelling, and lymphatic dysfunction [113, 114]. Elevated IFP hampers convection-driven transport, restricting oxygen and drug penetration and promoting hypoxia and therapeutic resistance [115, 116].

Reducing IFP through ECM-degrading enzymes or anti-angiogenic therapy has been shown to improve drug dispersion and treatment efficacy [117, 118]. IFP gradients also regulate IFV, cell migration, and immune trafficking, influencing cancer progression and therapeutic response [112]. Recognizing how IFP governs these fluid-mechanical processes is therefore vital for designing drug delivery strategies that either normalize or exploit interstitial pressure gradients to enhance tissue penetration and precision therapy [119].

The most significant pathological alteration in ISF dynamics occurs within the solid tumor microenvironment. Here, abnormal angiogenesis takes place which leads to structurally and functionally compromised blood vessels [120]. Coupled with a lack of functional lymphatic drainage and a dense, desmoplastic stroma, this results in interstitial hypertension, characterized by a significantly elevated IFP [121–123]. In a typical solid tumor, IFP is high and relatively uniform in the core, often approaching microvascular pressure, which effectively nullifies the hydrostatic pressure gradient necessary for drug extravasation from the blood vessels [103]. One study that has showed a deviation in ISF dynamics was conducted by Paudyal et al., where they showed that the extreme desmoplastic reaction that occurs in pancreatic ductal adenocarcinoma (PDAC) results in a particularly high IFP which makes it a challenging target for drug delivery. This study then suggested that longitudinal monitoring of IFP and velocity that has been estimated by computational fluid modelling (CFM) has shown promise as an early imaging biomarker to predict response to stereotactic body radiotherapy (SBRT), suggesting that changes in ISF dynamics precede morphological changes [123]. CFM has been successfully applied to map IFP and velocity heterogeneity in lymph node metastases of head and neck cancer and in non-small cell lung cancer brain metastases (LCBMs) [103, 124]. In LCBMs, objective response to stereotactic radiosurgery was associated with lower post-treatment IFP heterogeneity. This highlights that the spatial variation of ISF dynamics is a critical prognostic metric [124].

### Comparative ISF dynamics in healthy and pathological tissues

Key biochemical and biophysical differences in ISF dynamics between healthy and diseased tissues is important for therapeutic and diagnostic applications. Under physiological conditions, ISF maintains homeostasis through tightly regulated exchange processes that balance nutrient transport, waste removal, and immune surveillance [125]. Quantitative differences in ISF dynamics between healthy and diseased tissues are central to understanding disease progression, biomarker distribution, and therapeutic transport. IFP in many tissues, such as skin, intestine and lung, is slightly negative relative to atmospheric pressure (on the order of −4 to + 2 mmHg) under physiological conditions and thus tends to pull fluid into the interstitial space [61]. This low-pressure environment supports slow interstitial fluid velocities (on the order of 0.1–1.0 μm/s), which are critical for maintaining cellular homeostasis, nutrient exchange, and immune surveillance across tissue compartments [126].

In contrast, pathological tissues, including solid tumours, fibrotic organs, and chronically inflamed sites exhibit a coordinated shift across multiple ISF parameters, as summarized in Table 2. IFP in tumours and fibrotic tissues can rise dramatically, often reaching 10–40 mmHg due to excessive vascular leakage, extracellular matrix (ECM) deposition, and impaired lymphatic drainage. IFP values reaching 100 mmHg have also been observed in subcutaneous nodules in melanoma patients [127].This elevated pressure reduces transcapillary transport, promotes fluid retention, and creates steep pressure gradients at tissue margins, fundamentally altering solute and drug distribution.

**Table 2:** Comparative ISF dynamics in healthy tissue versus pathological tissue

Parameter	Healthy tissue	Diseased tissue (e.g., tumour, fibrosis, inflammation)	References
Biochemical Signalling molecules	Normal cytokine and growth factor levels	Elevated levels of cytokines and growth factor levels	[125]
Interstitial Fluid Pressure	Low to slightly negative maintains gentle outward filtration balanced by lymphatic drainage	Significantly elevated. The amount of fluid is greatly increased and poorly drained, resulting in an increase of the pressure in the diseased tissue	[136, 137]
Lymphatic drainage	Efficient clearance and maintains interstitial homeostasis	Lymphatic drainage is often impaired	[131, 132]
Interstitial fluid velocity (IFV)	Interstitial fluid velocity is very slow, which is crucial for cell health	Inflammation in tissues causes an increase in IFV	[134, 135]
Fluid-tissue Mechanical Interaction	Compliant tissue, balanced capillary-matrix stress	Stiffened tissue and solid	[133]

Biochemically, diseased tissues display markedly elevated concentrations of cytokines, chemokines, and growth factors (e.g., IL-6, TGF-β, VEGF), reflecting sustained inflammatory signalling and aberrant tissue remodelling [128]. These changes are not isolated to a single compartment but are observed across tumours, fibrotic tissues, and inflamed organs, underscoring the systemic relevance of altered ISF composition [129, 130]. Importantly, such biomolecular enrichment in ISF often precedes detectable changes in blood, reinforcing the diagnostic value of compartment-specific ISF analysis.

Lymphatic dysfunction represents another unifying feature across pathological states. Whereas healthy tissues maintain efficient lymphatic clearance that stabilizes ISF volume and composition, diseased tissues frequently exhibit collapsed, obstructed, or insufficient lymphatic networks [131, 132]. The resulting reduction in clearance capacity contributes to fluid accumulation, elevated interstitial pressure, and prolonged retention of inflammatory mediators and therapeutic agents.

From a biomechanical perspective, pathological tissue stiffening, driven by collagen cross-linking, ECM densification, and cellular contractility, further disrupts ISF transport. Increased solid stress compresses blood and lymphatic vessels, while reduced tissue compliance alters fluid-matrix interactions, collectively restricting convective flow and exacerbating hypoxia and inflammation [133]. Although local ISF velocity may increase in inflamed regions due to edema-driven pressure gradients, this flow is often spatially heterogeneous and poorly organized, leading to inefficient solute transport and uneven drug distribution [134, 135].

The parameters summarized in Table 2 illustrate that pathological ISF dynamics arise not from a single altered variable but from the coupled dysregulation of pressure, flow, biochemical composition, lymphatic function, and tissue mechanics across compartments. This integrated disruption positions ISF not only as a passive reflection of disease but as an active mediator of pathophysiology, with direct implications for biomarker discovery, drug delivery, and the rational design of ISF-targeted diagnostic and therapeutic strategies.

## Therapeutic applications of ISF: advances in drug delivery and precision medicine

Taken together, the fluid-mechanical principles and compartment-specific alterations in ISF dynamics establish the biophysical basis for why ISF could potentially serve as a sensitive reporter of local pathology. Variations in IFP, flow, extracellular matrix architecture, and lymphatic function influence the concentration, accessibility, and temporal evolution of biomarkers within the interstitium. These factors underpin the growing interest in ISF as a minimally invasive medium for infection detection, disease monitoring, and point-of-care diagnostics. Importantly, these same biophysical features also govern how therapeutic agents distribute and persist within tissues, highlighting ISF as not merely a passive reservoir of biomarkers but a dynamic interface for drug delivery and precision medicine. While many prior reviews have focused primarily on MN diagnostics or wearable ISF monitoring technologies, the present review uniquely synthesizes ISF transport physiology with mechanistically driven therapeutic innovations. Emphasis is placed on ISF dynamics, including computational modelling, simulated ISF systems, and IFP modulation, to establish a framework for rational design of targeted therapies.

ISF acts as the immediate microenvironment for cells, and its composition and physiological pressure are subject to significant alterations in pathological conditions such as tumors [138]. This dynamic nature of ISF, particularly the change during disease states, presents both considerable challenges and unique opportunities to develop targeted drug delivery systems and the advancement of precision medicine [76, 138–140]. In this pursuit, drug delivery systems are indispensable tools to enhance drug efficacy while minimizing side effects [141]. The integration of biomaterial-assisted therapies and nanotechnology has further propelled the evolution of drug delivery systems, paving the way for innovative solutions that can interact with or directly use ISF for more precise and personalized treatments [141]. A key challenge in drug delivery, especially within solid tumors, is the high IFP prevalent in the tumor microenvironment [142, 143]. This elevated pressure significantly impedes drug penetration and distribution, limiting therapeutic effectiveness. Furthermore, the ISF dynamics are intricately linked to tumor behaviour, including progression and drug resistance [144, 145]. Understanding ISF flow and transport is essential for effective drug delivery, particularly in solid tumors. ISF acts as a medium for both therapeutic agents and immune-modulating molecules. Its dynamics influence not only drug distribution but also the behaviour of immune cells within the tumor microenvironment [146]. High IFP in tumors hinders drug transport, while reducing IFP has been shown to enhance delivery and therapeutic response [144, 147]. Computational modelling and DCE-MRI have helped researchers simulate and monitor how interstitial flow impacts drug dispersion [108, 148]. Furthermore, ISF contributes to immune cell migration and signalling, which is significant in immunotherapy. Strategies like ultrasound-mediated IFP modulation and infusion-based approaches are being developed to exploit these fluid dynamics for more effective therapies [149, 150]. Computational models have also assisted with predicting nanoparticle behaviour under varying IFP conditions, showing how vascular normalization and fluid dynamics impact therapeutic outcomes [151, 152]. Hydrogel-based MNs and stimuli-responsive nanoparticles (e.g., pH- or temperature-sensitive) are among the most promising tools [153]. Anti-VEGF therapies have also proven useful in reducing IFP to facilitate nanoparticle delivery into hypoxic tumor regions, enhancing the efficacy of chemotherapeutics [154, 155].

Many advances have been made over the last decade investigating the therapeutic applications of ISF in drug delivery and precision medicine. By examining specific applications, it further highlights the potential of ISF-based approaches to transform targeted drug delivery and personalized medicine using MNs, microfluidics platforms, convention-enhanced nanomedicine, as well as stimuli- and fibre-driven modalities.

### MN-assisted ISF modulation for targeted transdermal drug delivery and monitoring

Microneedling is a minimally invasive technique that creates micro-channels in the skin using fine needles, significantly enhancing permeability, and enabling direct and efficient transdermal drug delivery into the ISF [5, 156]. Beyond delivery, MNs enable access to ISF sampling of biomolecules like proteins and metabolites that can reflect systemic conditions [157]. This makes ISF’s accessibility via MNs pertinent for continuous biomarker monitoring, especially when integrated with wearable biosensors for real-time health tracking and personalized medicine applications.

In the context of targeted therapy, particularly for solid tumors, microneedling can be tailored to enhance localized drug efficacy and reduce systemic exposure [158, 159]. The modulation of ISF flow via MNs can help overcome the barriers posed by high tumor IFP, leading to more effective drug therapy [144]. Advances in biomaterial engineering for MNs also allow for sustained drug release into ISF, potentially enabling prolonged therapeutic action with minimized side effects. Furthermore, recent work emphasizes the role of ISF to decode the tumor microenvironment and inform personalized treatment strategies [76, 139].

Building upon the role of MNs in ISF-mediated transport, recent advancements in hollow microneedle (HMN) systems have underscored the significance of ISF as an active modulator of transdermal drug kinetics [160]. Bhuimali et al*.* developed a computational model using verapamil as a representative drug to simulate ISF-driven transport through HMNs into viscoelastic skin. The model integrated ISF dynamics, convective flow, and both Fickian and non-Fickian diffusion, revealing that MN tip diameter and injection velocity critically influence drug distribution and bioavailability [160]. Higher injection velocities enhanced tissue concentrations, while narrower tips promoted systemic uptake. Although assuming Newtonian ISF behaviour limits applicability to heterogeneous tissues [161], the study provides valuable insight into ISF-drug interactions. Future work incorporating non-Newtonian fluid models, adaptive skin properties, and experimental validation could enhance translational relevance, paving the way for precision HMN platforms tailored to patient-specific transdermal therapy.

These reported studies position microneedling not merely as a permeability-enhancing tool, but as a controllable regulator of ISF-mediated transport. The therapeutic impact of MN and HMN systems arises from their ability to actively modulate local ISF flow, pressure gradients, and residence time-parameters that are particularly critical in pathological tissues such as solid tumors with elevated IFP. Computational and experimental evidence demonstrates that MN geometry and injection dynamics directly determine whether drug transport favours localized retention or systemic uptake. This establishes MN design as a tunable variable rather than a fixed delivery constraint. From a translational standpoint, the ability to couple MN-mediated delivery with ISF sensing enables adaptive, feedback-driven therapy. Future precision MN platforms will therefore derive value not from enhanced penetration alone, but from deliberate integration of ISF mechanics, tissue pathology, and patient-specific delivery objectives. However, despite significant progress, several limitations remain. MN systems typically access only superficial dermal ISF and therefore may not accurately reflect deeper tissue microenvironments. Furthermore, the relatively small sample volumes obtained may restrict multiplex biomarker analysis unless highly sensitive detection platforms are employed [162]. Addressing these limitations will require improved integration between extraction technologies, biosensors, and microfluidic analytical systems.

### Microfluidic platforms for precision drug delivery and ISF-guided therapeutics

**Fig. 3:**
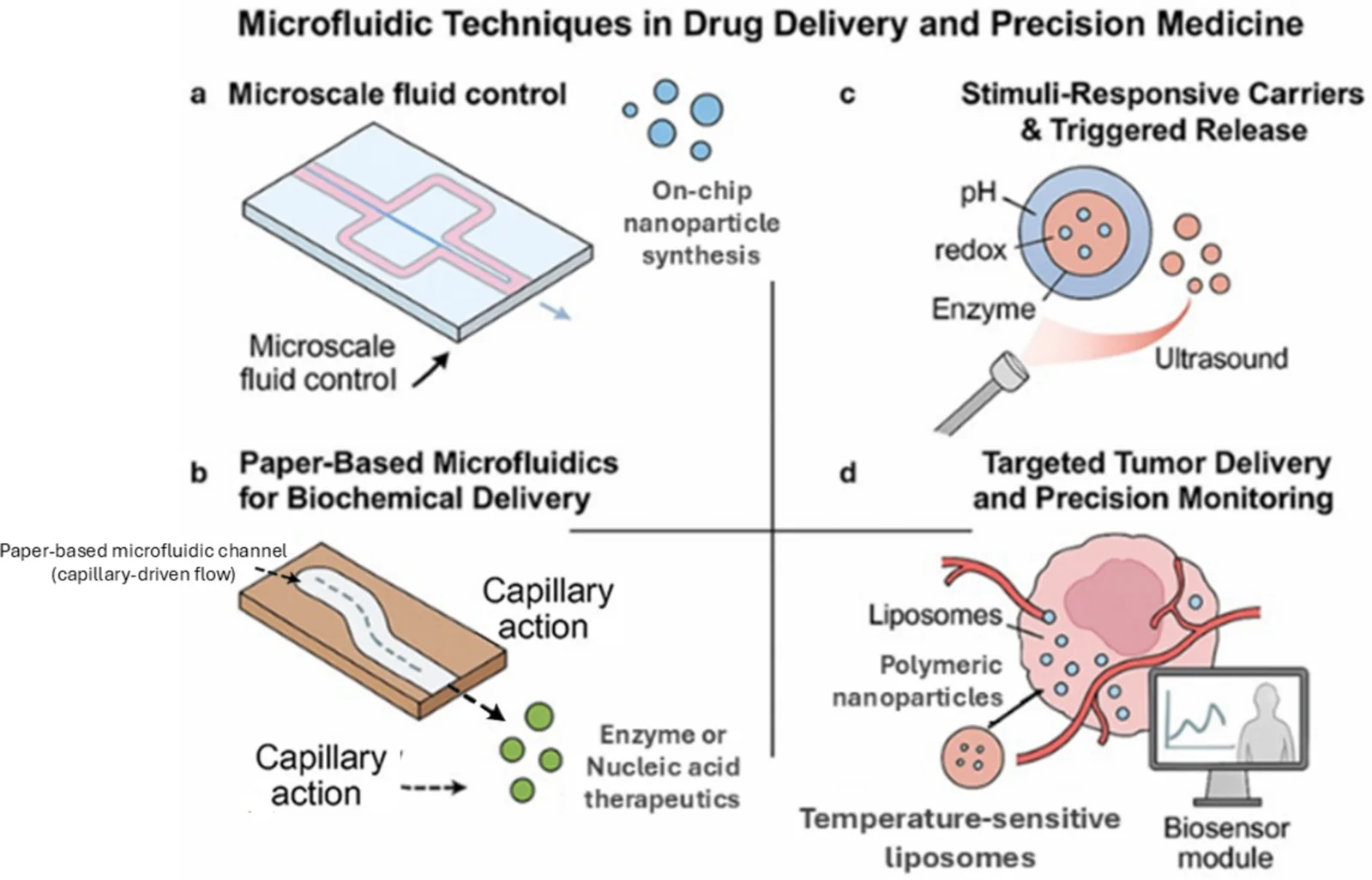
Microfluidic techniques in drug delivery and precision medicine. (**a**) Microfluidic microscale fluid control enables on-chip nanoparticle synthesis with tunable physicochemical properties for precise therapeutic applications. (**b**) Paper-based microfluidic devices (µPADs) utilize capillary-driven flow through patterned paper microchannels to deliver biochemical agents in a cost-effective and portable manner. (**c**) Microfluidic engineered stimuli-responsive carriers, including polymeric nanoparticles and temperature-sensitive liposomes, enable triggered drug release in response to physiological stimuli (pH, redox, enzymes) or external triggers such as ultrasound. (**d**) Microfluidic-assisted targeted tumor delivery improves drug transport through the interstitial space, while integrated biosensor modules enable real-time monitoring of therapeutic response, supporting precision medicine. (Created using images from Biorender.com)

Microfluidic techniques (Fig. 3) represent a promising approach for precision drug delivery; however, their translation into clinical practice remains limited by challenges such as device scalability, integration with biological tissues, and the complexity of reproducing physiological ISF dynamics in vitro, nevertheless, they represent a transformative approach in drug delivery and precision medicine, leveraging ISF for localized treatments like cancer therapy [163]. By providing precise control over fluid manipulation at the microscale (Fig. 3a), microfluidic devices facilitate the design of sophisticated drug delivery systems aimed at improving efficacy and reducing side effects [164].

Microfluidics enables the creation of novel drug delivery platforms with precise control over drug release rates, timing, and location. Examples include paper-based microfluidics (Fig. 3b) for efficient delivery of biochemicals and devices facilitating controlled release from temperature-sensitive liposomes using focused ultrasound, Fig. 3c [163]. Integrating microfluidics with nanotechnology allows for the production of nanoparticles with tailored properties, Fig. 3d [165]. This synergy facilitates the development of drug carriers with predictable and tuneable release characteristics, enhancing targeted therapy capabilities [166, 167]. Microfluidic techniques are used to fabricate carriers like liposomes and polymeric nanoparticles that can adapt their release profiles to the unique conditions within the tumor microenvironment, improving biodistribution and penetration through ISF. Advanced drug carriers leveraging stimuli-sensitive properties for robust targeted delivery can also be created using microfluidics. For instance, polymeric nanoparticles responsive to environmental stimuli have shown effectiveness in delivering agents to tumor sites, overcoming barriers like high tumor IFP [168, 169]. These platforms can be engineered to release drugs in response to specific biological signals (e.g., pH, redox conditions), maximizing therapeutic potential while minimizing systemic toxicity [170]. Moreover, microfluidic devices are advancing personalized medicine by enabling the real-time monitoring of drug effects and patient responses [171, 172].

These developments signify that microfluidic platforms function not simply as fabrication or delivery tools, but as control architectures that integrate ISF transport dynamics with drug release logic. Their translational value lies in the ability to systematically tune carrier size, responsiveness, and release kinetics to overcome ISF-mediated barriers such as elevated tumor IFP and heterogeneous diffusion pathways. By aligning microfluidic design parameters with pathological ISF conditions, these systems enable predictive rather than empirical optimization of drug delivery. Importantly, integration of real-time sensing within microfluidic frameworks supports adaptive, patient-specific therapy modulation. Thus, microfluidics advance precision drug delivery by converting ISF from a passive transport medium into an actionable therapeutic variable.

### Synergistic convection-enhanced drug delivery and nanomedicine: modulating ISF for optimized tumor targeting

Convection-enhanced delivery (CED) is an innovative strategy specifically employed for drug delivery in treating solid tumors. It leverages the pressure-driven convection of ISF and enhances the distribution and accumulation of anti-cancer drugs within the tumor microenvironment (TME), bypassing the limitations of passive diffusion [173, 174]. This approach achieves higher therapeutic concentrations at the target site and reduces systemic toxicity.

A primary advantage of CED is the ability to achieve uniform drug distribution within tumors by delivering drug directly into the interstitium using pressure gradients. Computational modelling has demonstrated the efficacy of CED to enhance drug penetration and therapeutic outcomes under various IFP conditions [173, 174], as well as optimization based on tumor architecture and IFP was explored for invasive treatments. Studies show CED can improve drug delivery by managing ISF turnover, which impacts binding kinetics for biologics. Faster target binding and more efficient responses are observed in tissues with high fluid turnover [175]. CED also maintains controlled IFP, ensuring sustained drug availability, critical to treat rapidly proliferating tumor cells often subject to elevated IFP[137].

Recent investigations highlight the synergy of CED with nanomedicine. Nanoparticles designed to enhance drug retention and modulate IFP can be administered via CED for improved penetration and efficacy [176, 177]. These nanoparticles are engineered to respond to TME stimuli, optimizing drug release. Furthermore, advancements in biodegradable polymeric nanoparticles administered via CED have also shown potential to enhance the overall treatment response in cancer [178, 179]. CED is also a valuable tool to overcome the physical barriers in larger tumors, often exhibiting altered pharmacokinetics and higher IFP hindering drug diffusion. By modulating ISF dynamics, CED can substantially improve drug transport with nanomedicine and maintain necessary therapeutic concentrations. Successful preclinical and clinical applications of CED are linked to improved tumor regression and patient outcomes, highlighting its potential role in targeted cancer therapies [180, 181]. However, physical resistance imposed by a dense extracellular matrix remains a significant barrier that cannot always be overcome by increasing fluid pressure alone.

While CED enhances convective transport by increasing the pressure gradient within tumours, a complementary approach focuses on reducing physical resistance to interstitial flow through enzymatic remodelling of the extracellular matrix. Enzymatic modulation of the extracellular matrix (ECM), particularly through hyaluronidase, has emerged as an important strategy to facilitate interstitial transport and improve drug penetration in solid tumours. Hyaluronidase degrades hyaluronic acid, a major contributor to ECM density and elevated interstitial fluid pressure (IFP), thereby reducing hydraulic resistance to fluid flow. This mechanism is conceptually complementary to CED: while CED increases the driving force for interstitial flow, hyaluronidase decreases the physical barriers restricting it. Preclinical and clinical studies have demonstrated that hyaluronan depletion enhances the penetration and distribution of diverse therapeutics, including liposomal doxorubicin and nanoparticles, by normalizing tumour pressure gradients and improving transvascular transport [182–185]. When combined with modalities such as anti-angiogenic therapies or immune checkpoint inhibitors, hyaluronidase-mediated ECM remodelling further enhances tumour perfusion and treatment response in ECM-dense cancers [186, 187]. Incorporating hyaluronidase into ISF-targeted drug delivery systems therefore represents a promising strategy to improve therapeutic accessibility within the tumour microenvironment.

The research discussed above shows that CED exemplifies a pressure-driven strategy that exploits ISF convection to overcome diffusion-limited drug transport. However, its therapeutic effectiveness is not determined by infusion pressure alone, but by the balance between convective driving forces and the hydraulic resistance imposed by the tumour ECM. The integration of CED with ECM-modulating approaches such as hyaluronidase highlights a transition toward transport co-optimization, where ISF flow is enhanced both by increasing pressure gradients and by reducing structural barriers. When combined with nanomedicine platforms engineered for retention and stimulus-responsive release, CED enables more predictable intratumoural drug distribution under high-IFP conditions. This systems-level perspective positions ISF as an active regulator of therapeutic kinetics, underscoring that durable improvements in tumour drug penetration will require coordinated modulation of ISF dynamics, ECM architecture, and carrier design rather than reliance on convection alone.

### Stimuli-driven drug delivery systems in cancer therapy: modulating ISF for enhanced tumor penetration

Electroosmotic flow (EOF) techniques use electric fields to control the movement of ISF and enhance drug delivery through biological tissues [188]. By interacting with the charged interfaces of porous tissues, EOF enables deeper penetration of drugs across the skin and tumor matrix. EOF can also significantly help reduce elevated IFP to improve drug distribution, when used alongside VEGF inhibitors to decrease tumor vascular permeability [154]. Biomaterials advancements have led to the development of biocompatible hydrogels and MN systems that can generate EOF for sustained and targeted drug delivery [189]. Moreover, EOF is increasingly used in microfluidic applications where electroosmotic pumps provide precise, programmable control over drug transport [190]. These tools are particularly valuable to deliver personalized therapies that consider patient-specific ISF dynamics and tissue characteristics.

Physical stimuli such as ultrasound can offer precise and non-invasive control over ISF dynamics. Acoustic cavitation and localized heating increase membrane permeability, thereby improving drug uptake in tumor tissues [191]. When combined with microbubbles, ultrasound enhances ISF flow and exploits the enhanced permeability and retention (EPR) effect for nanoparticle delivery. Ultrasound-responsive nanocarriers allow spatiotemporal control of drug release, advancing the goals of precision medicine [192]. Ultrasound imaging, especially when combined with Dynamic Contrast-Enhanced Magnetic Resonance Imaging (DCE-MRI), provides real-time feedback on ISF flow and drug distribution, which is critical to optimize treatment plans on a per-patient basis.

Pulsating heat sources also represent a novel approach to enhance drug delivery through ISF by reducing IFP and increasing vascular permeability [193]. Localized heating improves perfusion and facilitates drug and nanoparticle transport into the tumor interstitium.

Heat exposure also increases cell membrane permeability and can activate enzyme-catalyzed drug release from hydrogels. Thermosensitive nanocarriers that respond to heat stimuli can be co-administered for synergistic effects [194]. In addition to enhancing delivery, pulsating heat may also affect metabolic and signalling pathways in tumors, contributing to growth inhibition and improved drug efficacy [195, 196].

Electroosmotic, acoustic, and thermal stimuli constitute externally controllable strategies for actively modulating ISF transport and overcoming interstitial resistance in diseased tissues. Their primary advantage lies in programmability: electric fields, ultrasound, and localized heating can be precisely tuned in space and time to regulate permeability, IFP, and convective flow without permanently altering tissue structure. When combined with responsive biomaterials and real-time imaging feedback, these approaches enable adaptive control of drug distribution tailored to patient-specific ISF dynamics. Rather than acting as isolated delivery enhancers, physical stimuli function as regulatory layers that complement nanocarrier design, enzymatic ECM modulation, and biological targeting strategies. This integrated perspective reinforces ISF as a dynamic and manipulable therapeutic interface rather than a passive conduit for diffusion.

### Innovative fiber-based modalities for ISF-mediated drug release and sensing

**Fig. 4:**
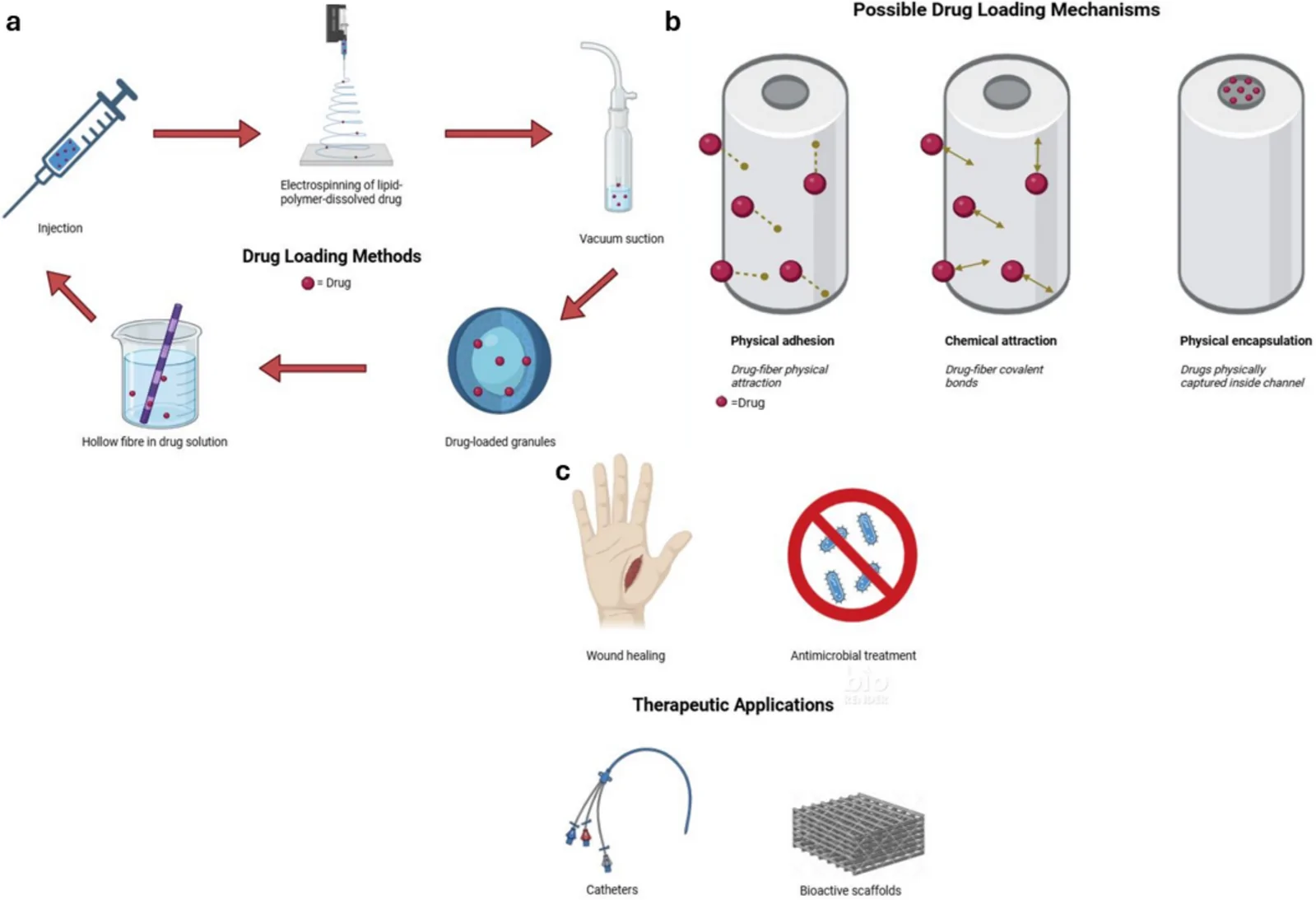
Schematic representation of drug loading methods, applications, and mechanisms in hollow fiber-based drug delivery systems. (**a**) Various drug loading techniques include injection or vacuum suction, dipping hollow fibers into drug solutions, dissolving drugs in liquid polymer and electrospinning, and incorporating drug-loaded core granules into the fiber structure. (**b**) Three primary drug-loading mechanisms: physical adhesion (via intermolecular forces), chemical attraction (via covalent bonding between drugs and fiber surfaces), and physical encapsulation (where drugs are confined within the fiber’s inner channel). (**c**) Broad biomedical applications of drug-loaded hollow fibers, such as in wound healing, antimicrobial treatment, catheter-based therapies, and bioactive scaffolds. (Created using images from Biorender.com)

Hollow fibers serve as efficient drug carriers owing to their high surface-area-to-volume ratio, tunable structure, and substantial loading capacity [197]. As summarized schematically in Fig. 4a, drugs can be incorporated into hollow fibers using multiple complementary approaches, including injection or vacuum-assisted suction into the lumen of the hollow fibers, immersion of fibers in drug solutions, electrospinning of drug-polymer mixtures, or incorporation of drug-loaded granules within the fiber core. These strategies allow precise control over drug localization within the fiber structure, which in turn governs release uniformity, stability, and duration. By adjusting wall thickness and cavity diameter relative to the drug’s diffusion properties, they enable controlled, sustained, and uniform release. Selectively permeable layers further regulate drug flow, while encapsulation within the fiber enhances stability and pharmacokinetic performance [197]. Release occurs through diffusion or capillary action.

The functional versatility of hollow fiber systems arises from the distinct drug-fiber interaction mechanisms illustrated in Fig. 4b. Drugs may associate with fibers through physical adhesion driven by intermolecular forces, chemical attraction via covalent bonding to functionalized fiber surfaces, or physical encapsulation within the inner channel. These mechanisms dictate release behaviour, with surface-associated drugs typically exhibiting faster diffusion-driven release, while chemically bound or encapsulated drugs enable prolonged and more controlled delivery [197]. Release can occur via passive diffusion across selectively permeable membranes or through capillary-driven transport along the fiber lumen. As shown in Fig. 4c, such tunability has enabled hollow fiber-based systems to be deployed across diverse biomedical applications, including wound healing, antimicrobial therapies, catheter-based drug delivery, and bioactive scaffolds for tissue regeneration. Hollow fiber-based systems have already demonstrated utility in localized drug delivery for conditions such as bacterial vaginosis and periodontal disease, and their potential continues to grow with advances like HDR-enabled delivery [197].

Hydrodynamically-driven release (HDR) using hollow fibres with high hydrodynamic permeability (HFHP) is an emerging and innovative drug delivery strategy that exploits the natural flow of ISF to enhance localized drug efficacy [198]. Unlike passive diffusion-driven systems, HDR employs convective transport, where ISF enters the fiber, dissolves encapsulated drugs, and carries the drug-rich fluid toward lymphatic targets. This mechanism enables release rates up to two orders of magnitude higher than traditional methods, particularly for macromolecules with limited diffusivity [198]. By sustaining near-saturated drug concentrations, HDR supports targeted therapies such as immunotherapy and metastasis suppression. However, the effectiveness of HDR depends on adequate ISF flow, which may be impaired in certain disorders, like Alzheimer’s disease [199]. Although reduced ISF flow may limit efficacy, multiplexed fiber configurations can mitigate dilution effects and maintain consistent release in low-flow conditions. Furthermore, integrating hydrodynamic vibration analysis into multiplexed systems (through both analytical and computational fluid dynamics (CFD) modelling) may optimize flow dynamics and shear rates, enhancing mass transfer and preventing drug dilution, thus advancing HDR toward precision medicine [200].

Efforts to expand advances in HDR systems include integrating ISF dynamics with fiber-optic theranostics (FOT), which introduces a powerful platform for simultaneous cancer diagnosis and therapy [201]. As demonstrated by Ran et al*.*, FOT employs minimally invasive fiber-optic needles implanted within tumors to exploit the biomarker richness of ISF. Functionalized fibers equipped with nitroreductase-responsive fluorescent probes detect hypoxia biomarkers directly in the tumor microenvironment, enabling precise, real-time mapping of malignancies without systemic agents needles [201]. Therapeutically, rare-earth doped fibers convert laser energy into localized heat for photothermal ablation, while embedded fiber Bragg gratings monitor temperature to prevent tissue damage. However, the platform’s reliance on sufficient ISF volume and hypoxia expression may restrict efficacy across variable tumor types and stages. Long-term biocompatibility and immune responses to fiber materials also remain underexplored. Furthermore, anatomical challenges in fiber placement, especially in mobile or deep organs, pose technical hurdles.

Although efficacy depends on tumor ISF levels, hypoxia extent, and biocompatibility, future refinements such as robotic guidance, broader biomarker detection, and long-term in vivo validation could overcome current limitations. Additionally, fiber-based ISF delivery and sensing platforms establish a quantitative and mechanistically unified framework in which drug transport, interstitial convection, and real-time feedback are co-engineered. Hollow fibers transition ISF from a passive diffusion medium to an active convective driver, particularly under HDR, where ISF flow rates directly determine drug flux, residence time, and lymphatic targeting efficiency. Importantly, these fiber systems integrate across compartments, namely, interstitium, lymphatics, and local vasculature, by coupling ISF flow with controlled release and downstream immune or metastatic pathways. The convergence of HDR with fiber-optic theranostics further extends this integration, enabling simultaneous sensing, actuation, and therapeutic response within the same ISF microdomain. While efficacy remains dependent on local ISF availability and tissue mechanics, advances in multiplexing, CFD-guided optimization, and minimally invasive placement position fiber-based ISF platforms as scalable, data-informed tools for precision drug delivery and localized disease monitoring.

### Simulated ISF systems as translational tools for next-generation therapeutic design

Simulated interstitial fluid (SISF) models have emerged as vital tools for bridging in vitro and in vivo drug performance, particularly for subcutaneous delivery where animal data may not predict human outcomes [202]. Torres-Terán et al*.* developed SISFs that mimic mouse-rat (SMR-ISF) and human-monkey (SHM-ISF) ISF compositions, replicating key physicochemical traits such as electrolyte balance, albumin content, and buffering capacity. Using liraglutide as a model drug, these systems revealed species-specific absorption linked to albumin binding performance [202]. SISFs provide reproducible, stable, and ethical platforms for long-acting formulation testing, offering mechanistic insight and translational value for next-generation subcutaneous therapies. Complementing experimental SISF models, Moghadam et al*.* developed a 3D computational framework to simulate ISF dynamics within tumors, providing critical insight into how interstitial parameters influence drug transport [203]. The model incorporates tissue permeability, tumor size, and lymphatic absence to predict IFP and IFV profiles. Results showed that larger tumors exhibit higher IFP, restricting drug penetration, whereas enhancing hydraulic conductivity improves distribution [203]. By accounting for necrotic and heterogeneous tissue structures, the model supports the design of tailored delivery strategies such as nanoparticle systems or localized injections. Ultimately, such simulations offer a predictive, non-invasive means to optimize personalized cancer therapies by pre-validating interstitial-targeted delivery approaches.

Extending SISF applications beyond oncology, a recent simulation study demonstrated its utility in optimizing drug-coated balloon (DCB) therapies for cardiovascular interventions [204]. Incorporating patient-specific arterial geometries and plaque heterogeneity, the model used Brinkman flow and advection-reaction–diffusion equations to capture drug–tissue interactions under interstitial fluid flow (IFF) therapy [204]. Findings revealed that IFF enhances drug residence time, binding, and penetration, particularly in calcified regions, underscoring the need to integrate fluid dynamics into DCB design for improved, patient-specific therapeutic precision. SISF applications have also been extended to the central nervous system, where a novel, dynamic 3D in vitro platform was developed to replicate IFF in glioblastoma multiforme (GBM) using a custom-designed perfusion bioreactor [205]. The system showed that higher flow rates (> 3 μL/min) markedly enhanced CXCL12 release from alginate/chitosan nanoparticle-loaded hydrogels, confirming convection as the dominant transport mechanism [205]. By closely mimicking the brain’s microenvironment, this physiologically relevant model enables optimization of convection-enhanced, localized drug delivery strategies for GBM and other CNS disorders.

#### Integrating ISF physiology and modelling for rational therapeutic design

Understanding the physiology of ISF provides the essential foundation for developing accurate computational and simulated models that inform advanced drug delivery system design. The Starling forces, capillary hydrostatic pressure, oncotic gradients, and lymphatic drainage, govern the bidirectional exchange of fluid between capillary and interstitial compartments. These forces establish the baseline fluid fluxes that computational frameworks aim to reproduce, thereby reflecting the physiological state of tissues [81, 206].

The structural elements of the extracellular matrix (ECM), including collagen fibres and hyaluronic acid, play a pivotal role in determining local hydraulic conductivity and resistance to interstitial flow. These parameters shape pressure gradients that drive solute transport and drug dispersion within tissues [159, 206]. In pathological conditions such as solid tumours or fibrotic lesions, ECM remodelling and elevated vascular permeability give rise to high IFP, which can severely hinder drug penetration and therapeutic efficacy. For instance, studies in pancreatic ductal adenocarcinoma have shown that enzymatic degradation of ECM components can mitigate these barriers and enhance intratumoral drug delivery [144, 207].

Incorporating such physiological determinants into computational models enables prediction of how disease-induced changes alter ISF dynamics and transport efficiency. Elevated IFP and increased ECM stiffness predictably reduce convective transport, diffusion, and overall drug dispersion, factors that can be integrated into simulation frameworks to optimize delivery strategies [208, 209]. By adjusting these parameters, researchers can identify approaches that either normalize interstitial pressure or exploit pressure gradients to improve drug accessibility. Therapeutic modalities that leverage convection-driven flow or enzymatic ECM modulation have demonstrated promise in enhancing intratumoral drug distribution [210]. Furthermore, incorporating disease-specific ISF compositions into formulation design ensures that ionic and protein environments are accurately represented, improving predictions of drug solubility, stability, and release kinetics [159, 209]. This synergy between ISF dynamics, ECM structure, and vascular architecture underpins the development of adaptive and predictive drug delivery platforms, maximizing efficacy while minimizing systemic exposure.

Beyond physicochemical modelling, the hydrodynamic behaviour of ISF also influences cellular activities such as migration, immune cell trafficking, and matrix remodelling. Computational frameworks that couple these biological and fluid-mechanical variables offer a more comprehensive understanding of therapeutic outcomes in complex microenvironments [117, 211].

### Harnessing tumor ISF dynamics for transcatheter and immunotherapeutic precision delivery

Transcatheter drug delivery, a targeted method involving the administration of drugs via a catheter into blood vessels, is widely used in cancer treatment [212]. However, its effectiveness is often limited by the tumor ISF environment, particularly the elevated IFP commonly observed in solid tumors [147, 213].

To enhance the effectiveness of transcatheter drug delivery, significant advancements have been made in the techniques employed and the materials used. Transcatheter arterial chemoembolization (TACE) is a well-established technique to treat liver cancer and has been extensively reviewed by Chen and coworkers [214]. Supporting this trend, recent research has reported the development of multifunctional microspheres that combine embolization with on-demand, temperature-triggered drug release. These microspheres allow simultaneous visualization of both the embolic material and the drug release process through dual MRI contrast agents [215]. Further considerations to enhance TACE techniques in cancer therapy could involves the development of nanoparticles specifically engineered to overcome the barrier of elevated IFP [216]. For example, studies have shown that fusiform-shaped nanoparticles, characterized by their elongated shape and high curvature, exhibit superior extravasation from blood vessels and penetration into tumor tissues compared to spherical or rod-shaped nanoparticles of similar chemical composition, even in the presence of high IFP [216]. This enhanced penetration leads to improved intratumoral drug accumulation and consequently, better antitumor efficacy [216]. The development of such novel nanoparticle designs, capable of navigating the challenging TME, represents a significant step towards improving the outcomes of transcatheter drug delivery to solid tumors.

Recent work has also highlighted the critical role of IFP in optimizing transcatheter intra-arterial (IA) drug delivery for solid tumors [217]. Using a VX2 liver tumor model, Liang et al*.* demonstrated that embolization with polyvinyl alcohol or Lipiodol reduced tumor IFP by up to 31.8%, significantly enhancing doxorubicin penetration [217]. These findings underscore the therapeutic potential of IFP modulation in improving intratumoral drug distribution, emphasizing the need to integrate tumor-specific biophysical profiling into personalized transcatheter treatment strategies.

**Fig. 5:**
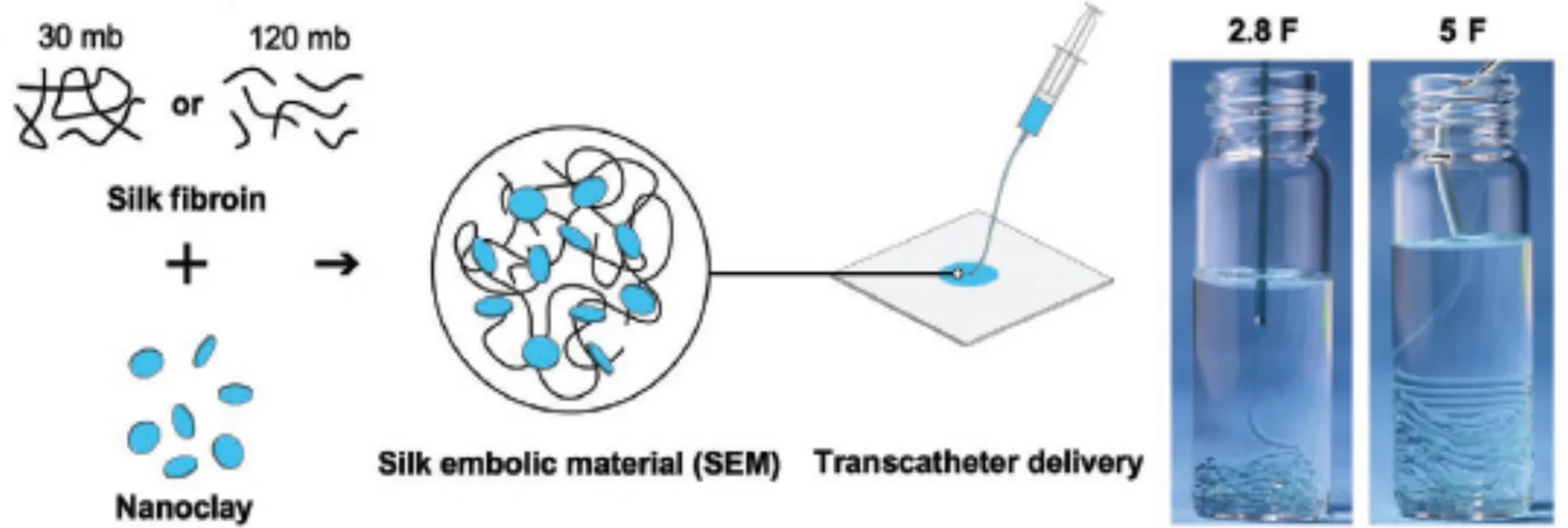
Schematic of silk embolic material and catheter-based drug delivery. Silk fibroin (30 or 120 kDa) is blended with nanoclay to form a silk embolic material, suitable for transcatheter administration. The material demonstrates injectability through catheters of various sizes (2.8 F and 5 F catheters) into aqueous environments. Reproduced with permission from [218]

Further scientific contributions in this space have introduced a novel silk-based embolic material that synergizes arterial embolization with sustained, ISF-mediated drug delivery, advancing precision transcatheter therapy **(**Fig. 5**)**. This dual-functional material not only occludes arterial blood flow but also acts as a localized depot for controlled drug release into the interstitial space [218]. Upon catheter-directed administration, the silk matrix both blocks blood flow and serves as a localized depot for controlled drug diffusion into the interstitial space. This dual mechanism leverages natural IFP gradients to enhance delivery of macromolecules such as Nivolumab (Opdivo)—an immune checkpoint inhibitor—achieving deep, sustained tumor penetration for over two weeks with minimal systemic toxicity [218]. Drug transport was further improved when arterial membrane disruption facilitated transvascular migration into tumor tissue [153]. Unlike conventional embolization or nanoparticle systems, this approach actively exploits ISF dynamics rather than merely mitigating IFP, enabling prolonged, localized release in solid tumors. Although further validation in human models is needed, silk-based embolic materials offer a promising, minimally invasive platform for personalized, site-specific cancer therapy.

ISF also shapes immune responses within tumors. Recent advances highlight the tumor interstitial fluid (TIF) as an active participant in immunotherapy, serving both as a delivery conduit and immune-activating niche [219]. A sodium alginate hydrogel co-loaded with low-dose doxorubicin and the immune adjuvant R837 enables localized, sustained release within the tumor interstitium, inducing immunogenic cell death and dendritic cell activation [219]. This ISF-mediated approach enhances immune precision, reduces systemic toxicity, and synergizes with checkpoint blockade (aPD-L1) to boost cytotoxic T-cell infiltration and macrophage polarization, establishing a foundation for localized, vaccine-like cancer immunotherapies [219]. This research highlights the underutilized potential of ISF not only as a passive transport medium but as a dynamic immunological interface. It further redefines ISF as an active immunological interface, enabling site-specific, vaccine-like cancer immunotherapies with enhanced precision and safety.

Further scientific contributions showed that hydrogel-based delivery systems, when injected intratumorally, can interact beneficially with the interstitial matrix [220]. These hydrogels enable sustained release of immune-stimulating agents, such as STING (Stimulator of interferon genes) agonists, which activate dendritic cells and promote durable, vaccine-like immune memory. However, hydrogel composition must be carefully optimized, as the interstitial immune milieu can elicit adverse immune reactions against the carrier itself [220]. Tumor stiffness and IFP critically influence therapeutic dispersion—softer tumors favour wider diffusion, while stiffer, high-pressure environments require adaptive delivery strategies for improved local efficacy [220]. While promising, the study’s limitations include unaddressed immune responses to hydrogels, tumor variability, and untracked IFP dynamics; future research should optimize formulations and delivery to fully harness ISF for precise immunotherapy.

These studies reveal that the success of transcatheter and intratumoral immunotherapies is governed less by delivery route alone than by the dynamic biophysical state of the TIF. Elevated IFP, ECM density, and spatial heterogeneity emerge as dominant regulators of drug penetration, residence time, and immune engagement. Notably, therapeutic strategies that actively modulate or exploit these parameters consistently achieve superior intratumoral exposure. Embolization-based approaches reduce IFP and re-establish pressure gradients, while shape-engineered nanoparticles and silk-based depots convert residual interstitial flow into a delivery advantage, enabling sustained macromolecule transport that diffusion-limited systems cannot achieve. Immunotherapeutic platforms further demonstrate that ISF is not only a transport medium but also an immunological regulator, where controlled interstitial release kinetics dictate antigen presentation, immune cell recruitment, and therapeutic synergy with checkpoint blockade. These findings collectively argue for a paradigm shift: effective transcatheter and immunotherapeutic design must be guided by tumor-specific interstitial biophysics rather than generic pharmacokinetic assumptions. However, translational gaps remain, including limited real-time IFP monitoring, incomplete characterization of patient-to-patient ISF variability, and insufficient integration of biomechanical metrics into treatment planning. Addressing these gaps through ISF-aware material design, biophysical profiling, and adaptive delivery strategies will be critical to realizing truly personalized, high-precision cancer therapies.

### Integrating ISF biomarkers with multi-omics approaches for individualized therapy

The integration of ISF analysis with multi-omics technologies such as proteomics, metabolomics, and cell-free DNA (cfDNA) sequencing, represents a transformative step toward precision and individualized medicine. ISF provides a localized snapshot of biochemical and physiological states at the tissue level, capturing dynamic molecular changes that are often diluted or masked in systemic circulation. Combining ISF-derived molecular profiles with high-throughput omics platforms allows for unprecedented resolution in disease characterization, drug response prediction, and therapy optimization.

Proteomic integration of ISF enables detailed mapping of signaling networks and pathway perturbations associated with disease. Recent advances in liquid chromatography-tandem mass spectrometry platforms, as well as data-independent acquisition (DIA) proteomics-based diaPASEF (data-independent acquisition with parallel accumulation-serial fragmentation) technologies have facilitated the detection of low-abundance proteins in ISF, including cytokines, growth factors, and post-translationally modified peptides that correlate with specific disease phenotypes [221–223]. For example, quantitative ISF proteomics from dermal and tumor tissues can distinguish between inflammatory, fibrotic, and oncogenic microenvironments, and also has significant implications for the clinical diagnosis of immunological diseases and skin infections, thereby informing individualized drug selection and dosing regimens [57, 224–226]. Integration with transcriptomic and single-cell data further strengthens precision mapping by linking local protein expression to gene activation states and cellular heterogeneity within diseased tissue.

Metabolomic profiling of ISF offers a complementary layer of insight into patient-specific metabolic reprogramming and therapeutic responses. ISF metabolites show significant correlations with plasma metabolites, while offering a minimally invasive approach that potentially enhances patient response and compliance [9, 43, 63]. Studies integrating ISF metabolomics with pharmacokinetic modelling have shown that small molecules, fatty acids, amino acids, nucleic acids and other relevant metabolites found in ISF can serve as valuable metabolomic biomarkers, which not only reflect underlying biochemical mechanisms but also provide crucial molecular insights for developing novel diagnostic tools and personalized therapeutic strategies [9, 63, 227, 228]. For example, in patients with atopic dermatitis treated with dupilumab, ISF sampling revealed baseline disturbances in amino-acid and lipid metabolism in lesional skin, followed by therapy-associated increases in long-chain fatty acids and changes in immune and microRNA markers, demonstrating how ISF metabolomics can sensitively detect local metabolic shifts linked to clinical intervention [229]. Recent work has further advanced ISF metabolomics by developing refined methods to collect ISF directly from intestinal mucosa through tissue centrifugation and elution, validated in rodents and successfully translated to human tissue [230]. These approaches enable direct quantification of microbiota-derived metabolites, cytokines, and proteins at their site-of-action, revealing that short-chain fatty acids such as propionate and butyrate are enriched in colonic ISF. Segment-specific profiling showed that ISF metabolite and cytokine concentrations were often significantly higher than in plasma, underscoring ISF’s ability to capture localized biochemical activity [230].

Collectively, these findings reinforce ISF as a highly informative biofluid for mapping local metabolic and immunological processes that are not apparent in systemic circulation. Integrating these datasets with systemic metabolomic and pharmacogenomic information could enable dynamic therapy adjustment—for instance, tailoring anti-cancer or anti-inflammatory dosing based on the evolving metabolic microenvironment of a tumor or lesion.

A particularly promising frontier lies in ISF-based cfDNA and microRNA sequencing, which can non-invasively reveal mutational and epigenetic landscapes at the site of pathology. cfDNA in ISF originates from apoptotic or necrotic cells within local tissues, providing higher spatial specificity than plasma cfDNA [231]. By integrating ISF cfDNA sequencing with tissue genomics and circulating tumor DNA (ctDNA) analyses, clinicians can differentiate localized tumor evolution from systemic disease progression, enabling more accurate therapy adjustments and monitoring of minimal residual disease [231]. Despite this conceptual promise, there remains a paucity of original research explicitly characterizing ISF-derived cfDNA. To date, no well-documented studies have applied next-generation sequencing or fragmentomic profiling directly to ISF samples in this context. Most reviewed literature highlighted the diagnostic potential of cfDNA recovered from “non-blood” biofluids but did not specifically address ISF as an analyte reservoir [232, 233]. For instance, recent studies using bronchoalveolar lavage (BAL) provide valuable insights into the diagnostic potential of locally derived cfDNA. The analysis of cfDNA analysis from BAL fluid identified mutations matching those in primary lesions in 93.8% of cases, far outperforming both plasma cfDNA and cytology-based approaches [234]. This finding highlighted the abundance of cfDNA within proximal microenvironments, reinforcing the concept that fluid compartments closely associated with diseased tissue harbor richer molecular signals than systemic circulation [234]. Expanding the spectrum of localized biofluids for precision oncology, tumor in situ fluid (TISF)—the fluid accumulated within the surgical cavity following glioma resection—has emerged as a novel and informative source of cfDNA [235]. In a retrospective study involving 10 glioma patients, cfDNA extracted from TISF revealed at least one tumor-specific mutation in all cases. Deep sequencing showed that TISF-derived ctDNA captured a wide array of clinically relevant mutations reflecting glioma heterogeneity, including alterations in PI3K/AKT/mTOR, tumor suppressor, and cell cycle pathways [235]. The ability of TISF to mirror the evolving genomic landscape of tumors underscores the diagnostic and prognostic value of locally derived cfDNA reservoirs.

These observations strengthen the premise that localized fluid compartments proximal to tumor sites, such as ISF, may harbor richer, temporally dynamic molecular information than systemic biofluids. Clinical adoption of ISF cfDNA analysis could thus enable real-time monitoring of tumor evolution, resistance mechanisms, and therapeutic responses with minimal invasiveness, advancing the frontier of personalized cancer management.

Similarly, microRNAs and ISF-derived microRNAs have been linked to hypoxia, angiogenesis, and immune modulation, providing both diagnostic and predictive value in cancer and metabolic disorders [78, 236–238]. For instance, microRNA profiling from breast tumor interstitial fluid (TIF) has provided original evidence that locally derived ISF fractions can reveal disease-relevant molecular signatures. In a study analyzing matched TIF, normal ISF, tumor tissue, and serum from breast cancer patients, 266 microRNAs were elevated in TIF compared to normal counterparts. Among these, several correlated with immune cell infiltration, adipocyte content, and patient survival, underscoring their dual role as mediators of tumor-stroma communication and as potential noninvasive biomarkers [76].

The evidence reviewed here demonstrates that ISF is not merely a surrogate for plasma, but a functionally distinct molecular compartment that captures biochemical, metabolic, and genetic states driving disease behaviour and therapeutic response. Proteomic and metabolomic analyses consistently show that ISF enriches for low-abundance, short-lived, and microenvironment-specific signals that are frequently diluted or undetectable in blood, enabling earlier detection of pathway activation, immune modulation, and metabolic reprogramming. When integrated with transcriptomics and single-cell data, ISF-omics offers a mechanistic bridge linking molecular signals to cellular heterogeneity and tissue-level function.

Importantly, the emerging data on ISF-derived cfDNA and microRNAs suggest that localized interstitial compartments may outperform plasma for tracking tumor evolution, resistance, and minimal residual disease. However, the current literature remains skewed toward proof-of-concept diagnostics, with limited standardization of ISF collection, sparse longitudinal datasets, and minimal integration into therapeutic decision frameworks. This represents a critical translational bottleneck. Moving forward, the true value of ISF-multi-omics will depend on its incorporation into adaptive treatment algorithms, where dosing, modality selection, and combination strategies are dynamically adjusted based on evolving interstitial molecular states rather than static baseline biomarkers. Achieving this will require harmonized sampling technologies, robust bioinformatic pipelines capable of integrating local and systemic data, and prospective clinical studies linking ISF-omics readouts to outcomes. If these challenges are addressed, ISF-centric multi-omics has the potential to transform precision medicine from descriptive molecular profiling into a predictive, feedback-driven therapeutic strategy grounded in the biology of local tissue microenvironments.

## Challenges and future perspective

Despite its immense potential, the clinical integration of ISF-based diagnostics and therapies faces significant challenges. A primary issue is the heterogeneous composition and variability of ISF across anatomical sites, individuals, and disease states, which complicates standardization. Moreover, ISF extraction remains limited by accessibility, particularly in deep or sensitive tissues like the brain or tumors with elevated IFP, which can hinder both sampling and therapeutic penetration. High IFP in solid tumors, for example, creates a hostile microenvironment that impairs drug delivery, posing a critical barrier to treatment. Another major limitation is the lack of robust clinical data and regulatory frameworks to support widespread adoption of ISF-based technologies. While MNs and wearable sensors have advanced significantly in preclinical studies, real-world validation and longitudinal studies in humans are scarce. Additionally, integration with existing healthcare infrastructure and digital health platforms remains underdeveloped. Future advancements must prioritize the development of adaptive, patient-specific ISF monitoring tools and biomaterial innovations that accommodate inter-individual variability. Continued interdisciplinary efforts are needed to refine computational models, simulate ISF dynamics, and optimize drug delivery systems under varying physiological conditions. Advances in multiplexed sensing, machine learning-driven diagnostics, and minimally invasive hardware will be instrumental in overcoming current limitations. Looking forward, expanding ISF applications into neurological, autoimmune, and paediatric conditions, while establishing regulatory pathways and safety standards, will determine the trajectory of integration ISF sampling as a mainstream clinical care tool. With these strategies, ISF could revolutionize personalized healthcare by enabling non-invasive, responsive, and precise diagnostic and therapeutic interventions.

## Conclusions

ISF holds tremendous potential as a versatile platform to advance precision diagnostics and targeted therapeutics. As a richly informative and locally representative biofluid, ISF provides real-time insights into tissue-level pathophysiology that blood-based analysis often overlooks. From minimally invasive MN-based biomarker detection to advanced drug delivery systems such as hollow fibers, convection-enhanced devices, and electroosmotic platforms, ISF is increasingly being recognized as a critical component in personalized medicine. Its dynamism and role in diagnosing or treating conditions like cancer, metabolic diseases, and neurodegeneration underscore its relevance. Simulated ISF systems and computational models help bridge the translational gap, facilitating drug formulation testing and optimization in preclinical stages. While current ISF-based technologies have shown strong proof-of-concept results, the road to clinical translation requires overcoming key hurdles, such as extraction standardization, IFP management, and regulatory validation. Nevertheless, the convergence of biomaterials science, bioengineering, nanomedicine and data-driven medicine is driving innovative solutions that may soon position ISF as a clinical mainstay. The integration of ISF into wearable devices, theranostic platforms, and immunotherapy further highlights its role as a biological interface that can enhance both monitoring and treatment.

Despite this growing momentum, the application of ISF in infectious disease diagnostics and therapeutics remains notably underdeveloped. Given its rich content of cytokines, antibodies, pathogen-specific proteins, and cfDNA, all key mediators of host–pathogen interactions, ISF offers a largely untapped opportunity to revolutionize infection monitoring and intervention. The evidence presented in this review, including novel MN sensors and biomarker-rich ISF signatures, provides a compelling foundation for future studies aimed at integrating ISF into infectious disease research, diagnostics, and personalized treatment strategies.

Ultimately, ISF offers a promising frontier for next-generation healthcare, enabling patient-specific monitoring, therapy optimization, and a paradigm shift toward more localized, real-time, and minimally invasive medical interventions.

## Data Availability

The datasets generated during and/or analysed during the current study are not available from the corresponding author but can be accessed from the cited work.

## Declarations

### Animal studies

Not applicable to the study.
